# DockBind: A Quality-Controlled Data Set of Modeled Protein–Ligand Complexes Associated with Affinity Data

**DOI:** 10.1021/acs.jcim.6c02093

**Published:** 2026-10-26

**Authors:** Alberto Rovetta, Alberto Memè, Hanne S. Antila, Ruth Brenk

**Affiliations:** † Department of Biomedicine, University of Bergen, Jonas Lies vei 91, 5020 Bergen, Norway; ‡ Computational Biology Unit, University of Bergen, Thormøhlensgate 55, 5008 Bergen, Norway; § Fachbereich Physik, Freie Universität Berlin, Arnimallee 14, 14195 Berlin, Germany

## Abstract

Ligand–protein interaction prediction is essential for structure-based drug discovery, but further development of machine-learning scoring functions is limited by a lack of high-quality training data sets. Here, we present DockBind, a curated data set of modeled protein–ligand complexes paired with experimental binding affinity data, generated through a quality-controlled docking workflow. This workflow was developed based on an expanded docking benchmark, where we systematically evaluated free and template-based docking methods as well as ligand similarity and maximum common substructure (MCS)-based pose filtering to generate and identify the most realistic poses. The resulting workflow was then incorporated into a pipeline integrating structural and binding affinity data from Binding MOAD, the Protein Data Bank, ChEMBL, and BindingDB, which resulted in 18,332 complexes across 612 protein targets, with high structural reliability and chemical diversity—the DockBind data set—providing a valuable resource for training and benchmarking machine-learning scoring functions.

## Graphical abstract

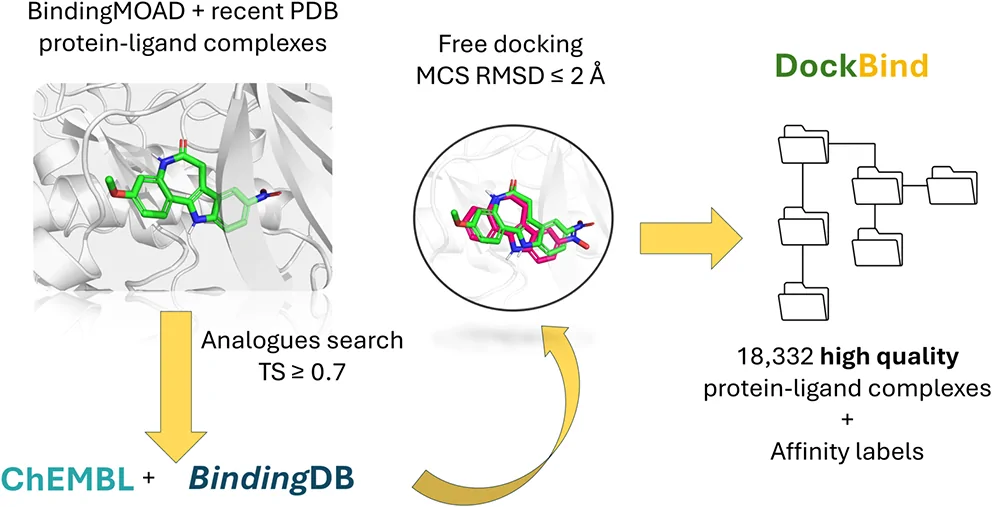

## Introduction

Quantifying protein–ligand interactions is central in understanding protein function and consequently to modern structure-based drug design, where computational approaches have become integral to hit identification and hit-to-lead optimization workflows.^1–3^ Among the range of methods available, molecular docking remains one of the most used for modeling protein–ligand complexes because it enables the rapid screening of very large compound libraries at comparatively low computational cost. Docking typically involves two steps: generation of ligand poses (conformations) for a ligand binding within a predefined binding site followed by prediction of an activity value using a scoring function.^4^ This allows potential binders to be distinguished from nonbinders in very large libraries of millions of compounds, without the need to synthesize and test them in the lab. Despite its widespread application, docking still suffers from key limitations including inaccurate binding mode prediction, insufficient treatment of protein flexibility and solvation effects, and limited scoring accuracy.^5,6^

Among the approaches used to refine the performance of docking simulations is template-based docking.^7^ Based on the assumption that similar ligands adopt similar binding conformations,^8^ template-based docking allows one to enhance docking simulations by placing the ligand in the target binding site using information from a crystal structure of the target protein in complex with a related ligand. According to recent studies, the choice of the template appears crucial for the success of template-based docking, with the chemical similarity and maximum common substructure (MCS) coverage playing a major role.^7,9,10^ However, to the best of our knowledge, a comprehensive analysis of the most suitable cutoff values for template selection has not yet been reported in the literature.

Further, the reliability of molecular docking heavily depends on the accuracy of the scoring methods used within the docking algorithm to estimate the binding affinity and predict the binding pose.^11,12^ These have well-known deficits particularly when estimating the binding affinity across diverse targets, resulting in false positive and false negative predictions. Machine learning scoring functions have emerged as a promising solution to address this problem.^13–17^ However, this type of scoring functions still exhibits limitations, particularly in terms of accuracy and generalizability on previously unseen protein targets.^18,19^ One of the major factors constraining progress is the quality and composition of the data sets used for model training and benchmarking. The data sets frequently contain hidden biases that affect performance, such as analogue, domain, and noncausal biases, and artificial enrichment, which can inflate apparent model performance while limiting real-world applicability.^20,21^

Efforts have been made to reduce these biases, for example by increasing chemical diversity in the training data set or increasing the number of active and inactive compounds.^22–26^ However, the scarcity of experimentally determined binding conformations remains a major limitation: in typical drug discovery projects, binding affinities for several compounds are measured, yet only a small subset are cocrystallized with the protein target to determine the binding pose of the ligands within the binding site. This creates a large gap in the data volume available for experimental binding affinities compared to the experimental protein–ligand complex structures. Public databases such as ChEMBL^27–29^ and BindingDB^30^ contain a wide range of affinity data without associated binding conformations while experimentally resolved complexes are primarily available through the Protein Data Bank (PDB).^31^ Through combining affinity data and structural data, popular data sets like PDBBind^32^ and Binding MOAD^33^ were created. The publicly available versions of these data sets contain about 23,000 and 15,000 complexes with affinity data, respectively. However, Binding MOAD is no longer maintained and the newest version of PDBBind is no longer freely available, freezing the openly accessible versions at the state of the PDB at 2020.^34^

To close the data gap between available affinities and experimental protein–ligand complex structures, one can generate binding conformations through *in silico* methods, such as molecular docking and comparative structure modeling.^9,21,35,36^ Like in template-based docking, this data is commonly generated under the assumption that similar ligands bind to the same target in a similar manner. Accordingly, the main filtering criteria for accepting or rejecting a computationally generated pose is typically the MCS root-mean-square deviation (RMSD) relative to a crystallographic reference ligand with high Tanimoto similarity based on different fingerprints (generally >0.6 or >0.7). However, although these criteria are widely used, systematic validation or quality control of the selection thresholds is often limited.^37^

The above-mentioned considerations have inspired us to build DockBind, a curated data set featuring quality-controlled computationally generated poses with their associated experimental affinity data. To generate DockBind we first constructed a benchmark data set using a subset of ligands from Binding MOAD based on the Astex Diverse set targets.^38^ Using this benchmark, we explored the impact of ligand similarity, template selection, and MCS RMSD-based pose filtering on docking success rates to establish optimized criteria for high-quality pose generation. This created the basis for an automated, benchmarked workflow used to generate the conformations in DockBind and the lesson learned in this exploration can be used more broadly as guidelines for template selection in template-based docking. We then enriched the Binding MOAD data set with structures from the PDB that were released after the last Binding MOAD update to obtain a list of potential docking templates, which were then used to select ligands with confirmed binding affinities from ChEMBL and BindingDB. Finally, the workflow established in the benchmark set was applied to generate binding poses for the selected ligands. The resulting DockBind data set contains 18,332 modeled protein–ligand complexes across 612 targets generated using a validated and quality-controlled workflow. By prioritizing structural reliability and chemical diversity, DockBind provides a valuable resource for training and benchmarking machine-learning scoring functions.

## Material and Methods

All scripts to evaluate docking, establish a workflow that ensures the generation of high-quality docking poses, and develop the DockBind data set were written in Python 3.10.16 with the help of the following libraries: RDKit 2024.09.3 (RDKit: Open-source cheminformatics. https://www.rdkit.org), Spyrmsd 0.8.0,^39^ Scikit-learn 1.3.2,^40^ Pandas 2.2.2,^41^ Numpy 1.26.4,^42^ and Matplotlib 3.9.3.^43^

### Validating Free and Template-Based Docking

#### Enriching the Astex Diverse Set to Generate a Validation Set

To explore the impact of different docking strategies and selection criteria on the quality of docked pose, we assembled a validation set of experimental protein–ligand crystal structures and then proceeded to perform docking on this set utilizing the crystal structure as ground truth. The validation set was constructed by enriching the Astex Diverse set^38^ with complexes from Binding MOAD v2020 ^33^ to increase the number of experimentally determined protein–ligand complexes available for docking validation. The Binding MOAD set was downloaded, and complexes that contained ligands with a 3-letter HET code and had a resolution ≤ 2.5 Å were selected, discarding complexes containing small peptides, oligonucleotides, or other nonsmall-molecule ligands in the binding site. PDB identifiers from both the Astex Diverse set and the filtered Binding MOAD data set were mapped to UniProt accession numbers using EMBL-EBI resources.^44^ For each target represented in the Astex Diverse set, all corresponding protein–ligand complexes from the filtered Binding MOAD sharing the same UniProt accession number were retrieved and incorporated into the benchmark set.

#### Structure Preparation

Ligands in the asymmetric unit of each structure were evaluated for interactions with symmetry mates generated using the PyMOL open-source package 3.1. Symmetry mate chains containing atoms within 5 Å of the reference ligand were retained to preserve relevant binding-site interactions for docking.

Structures belonging to the same UniProt accession number were subsequently aligned with PyMOL using the corresponding structure in the Astex set as template. Structures with an alignment RMSD ≥ 2.5 Å to the reference structure were discarded. Ligands were then extracted from the PDB files and saved individually in the SDF format. Bond orders were assigned with the Chem.AssignBondOrdersFromTemplate function available in RDKit using the ligand SMILES annotations stored in the PDB as templates.

#### Molecular Docking

Free and template-based docking was carried out using FlexX 5.2.^45^ The binding sites were automatically defined by the software based on the ligand contained in the crystallographic structures. In template-based docking, this reference ligand was additionally used as template to guide pose generation, whereas free docking was performed without positional constraints. A maximum of 10 poses were generated in each docking run. The resulting poses were optimized and rescored using HYDE 1.5 to estimate the upper and lower affinity limit; the upper affinity limit was used for pose selection and will be referred to as HYDE score in this work.^46,47^

The quality of the docking poses was evaluated by calculating the RMSD between the docked and crystallographic ligand using SpyRMSD 0.8.^39^ Poses with an RMSD ≤ 2 Å to the crystallographic reference pose of the molecule being docked were considered native-like. The docking power, also referred to as success rate, was then calculated as the percentage of protein–ligand pairs for which a native-like pose was predicted relative to the number of protein–ligand pairs for which at least one pose was predicted.

#### Maximum Common Substructure RMSD and Tanimoto Similarity

As filtering metrics, we explored MCS RMSD and Tanimoto similarities between the docked ligand and the reference or template ligand binding to the same pocket.

The MCS between the reference/template ligand and the docked ligand was determined using the RDKit rdFMCS.FindMCS function. The atom matching was based on element type (rdFMCS.AtomCompare.CompareElements) while exact matching was required for the bond order comparison (rdFMCS.BondCompare.CompareOrder), ensuring that only atoms of the same element and bond order were treated as equivalent. The RMSD between the MSC atoms of the two compounds was computed with SpyRMSD.

The Tanimoto similarity of two ligands was calculated with the DataStructs.TanimotoSimilarity function based on Morgan fingerprints. The Morgan fingerprints were generated as bit vectors with the RDkit AllChem.GetMorganGenerator and the GetFingerprint functions, using a radius of 2 and a bit size (nBits) of 2048.

### Assembling and Analyzing DockBind

#### Enriching Binding MOAD with Recent PDB Structures

Because the latest Binding MOAD release dates from 2020,^33,34^ we enriched the database with structures from the PDB to obtain an updated list of template protein–ligand complexes. X-ray structures released between January 2021 and June 2024 containing a ligand and having a resolution ≤ 2.5 Å were retrieved from the PDB and linked to BindingDB^30^ and ChEMBL^27^ entries through UniProt accession mappings provided by EMBL-EBI. Structures containing ligands with a Tanimoto similarity ≥ 0.7 to at least one ligand with an assigned binding affinity in BindingDB or ChEMBL were selected and downloaded from the PDB. Ligands were subsequently extracted from the PDB files following the procedure described above.

#### Assessing Docking Performance on Newer PDB Structures

To assess the performance of the docking pipeline on newer PDB structures, the structures used to enrich Binding MOAD were filtered to create a validation set distinct from the enriched Astex set. To avoid redundancy, when the same ligand was present in multiple structures of the same target, only the PDB entry with the highest crystallographic resolution was retained. Additionally, complexes containing covalently bound ligands were excluded, and targets represented by less than two distinct ligands were discarded to retain only the ones suited for a cross-docking experiment. Finally, targets included in the enriched Astex set were excluded.

Free docking was carried out as described above. Subsequently, the MCS RMSD between each docked pose and the corresponding crystallographic reference ligand in the receptor used for docking were calculated as described above. Poses with an MCS RMSD ≤ 2 Å were retained. When multiple poses for the same ligand–receptor pair satisfied the MCS RMSD cutoff, the pose with the best HYDE score was selected. When multiple reference ligands were available for the same ligand for the same target and yielded poses meeting the MCS RMSD cutoff, only the pose generated with the receptor of the reference ligand with the highest Tanimoto similarity was retained.

Success rates were calculated as described above and 95% confidence intervals were estimated using a nonparametric cluster bootstrap with UniProt targets as the resampling unit.^48^ In each of 10,000 bootstrap replicates, targets were sampled with replacement, and all accepted cross-docking pairs associated with each sampled target were retained.

#### Selection of Ligands to Dock from BindingDB and ChEMBL

Ligands for docking were retrieved from ChEMBL and BindingDB when a compound with a similar chemical structure was present in the enriched Binding MOAD data set. For the targets in the enriched Binding MOAD data set, ChEMBL binding affinity data were extracted from the ChEMBL 34 release hosted in an in-house MySQL database using the filters listed in Table 1. BindingDB activity data were downloaded directly from the BindingDB Web site without additional filtering.

**Table 1:** Filters Used to Extract Binding Affinity Data from ChEMBL

Condition	Filter	Comment
**assays.confidence_score**	≥8	Include only homologous single protein target assigned or direct single protein target assigned
**molecule_dictionary.chirality**	≠ 0	Avoid the inclusion of racemic mixtures measurements
**activities.data_validity_comment**	‘Manually validated’ or ‘Null’	Avoid inclusion of ‘problematic’ measurements – such as potential missing data, author or transcription error, or measurements outside typical range.
**activities.standard_type**	IC_50_ or *K*_d_ or *K*_i_	Include only activity types of interest

For each reference ligand in the enriched Binding MOAD data set, the Tanimoto similarity values were calculated against all the ligands with recorded activities for the same target in ChEMBL or BindingDB using Morgan topological fingerprints as described above. Only reference-ligand pairs with a Tanimoto similarity ≥ 0.7 were retained.

Three-dimensional ligand conformers for the docking were generated from the corresponding SMILES strings using RDKit: explicit hydrogens were added with the AllChem.AddHs function and conformers were generated with EmbedMolecule followed by geometry optimization using AllChem.UFFOptimizeMoleculeConfs. Default parameters were used for this process, except that the maximum number of iterations in the optimization step was increased to 10,000 to improve convergence for large or highly flexible molecules. The generated conformers were saved in SDF format, while ligands that failed this process were discarded.

#### Pose Generation and Binding Affinity Processing

Free docking with Tanimoto similarity based reference ligand selection and MCS RMSD postfiltering was carried out as described under “Assessing docking performance on newer PDB structures”. All complexes retained in this way were annotated with affinity data. When affinity data for the same compounds was contained in BindingDB and ChEMBL, only the data from ChEMBL was retained. When multiple binding affinity measurements of the same type for a given target-ligand were available, the median value was retained for each activity type.

#### Deduplication

Redundant entries were removed from DockBind through a postdocking deduplication workflow. All docked compounds linked to the same reference ligand were compared, as distinct database entries might converge to the same chemical structure only after ligand preparation and docking. For this purpose, standard InChIKeys were generated from the SD files generated by HYDE with RDKit using rdkit.Chem.inchi.MolToInchiKey. Structures were considered equivalent according to Standard InChI normalization, which collapses differences in protonation, tautomeric, and Kekulé representation while preserving encoded stereochemical distinctions. For duplicated ligands, it was checked if the number of defined stereocenters in ChEMBL and BindingDB matched the number of stereocenters of the docked ligand and entries with fewer explicitly defined stereocenters were removed. For the remaining duplicated ligands the salt/free-form relationships was checked by identifying disconnected components in the parent SMILES. For cases containing one free-form entry and one salt form, the salt entry was removed. Affinity annotations from the removed salt record were only transferred to the corresponding free-form entry when it did not already include a value for the same affinity type. Duplicated ligands not resolved by these automated rules, including internal BindingDB redundancies, cyclohexane stereochemical artifacts, and other ambiguous cases, were manually curated.

#### Protein Family Representation

Protein family representation was assessed using Pfam annotations^49^ mapped to the experimental PDB structures through SIFTS.^50^ Multiple Pfam entries were allowed when more than one domain was present in the structure, but when calculating the cumulative coverage of the most represented Pfam entries, complexes associated with multiple annotations were counted only once.

#### Chemical Diversity Visualization

The chemical diversity of the resulting data set was analyzed using molecular fingerprints and dimensionality reduction. Ligands were first converted into Morgan fingerprint representation with the RDKit AllChem.GetMorganGenerator and the GetFingerprint tools as described above.

Uniform Manifold Approximation and Projection (UMAP)—a nonlinear dimensionality reduction technique that preserves both local and global structure in high-dimensional data^48^—was applied to the Morgan fingerprints to obtain a 2D representation of the ligand data set for visualization. The UMAP embeddings were generated with the UMAP and fit_transform functions implemented in the umap package using random_state = 42. The first and second components (UMAP-1 and UMAP-2) of the resulting embeddings were used to visualize the distribution of ligands from different data sets.

#### Analogue Bias and Scaffold Redundancy Analysis

Chemical series were identified independently for each target as described previously.^51^ In brief, compounds were represented using Morgan fingerprints (radius 2, 2048 bits), and pairwise Tanimoto distances were used for average-linkage hierarchical clustering. Candidate clusters were evaluated using their MCS and retained as chemical series when the MCS occurred in less than 0.1% of compounds in ChEMBL. No series was assigned to a target if all compounds shared only generic MCSs that occurred too frequently in ChEMBL to meet the series definition or if all ligands were classified as singletons.

Scaffold representation across the complete data set was assessed using Bemis-Murcko scaffolds calculated from the docked ligand structures.^52^ Compounds without a defined Bemis-Murcko scaffold were classified as acyclic and excluded from the count.

#### Validity Assessment of Docking Poses

For each protein–ligand complex in the final DockBind data set, the generated ligand pose was evaluated using PoseBusters.^53^ A pose was considered valid only when all 22 tests were passed. Poses failing one or more tests were retained in the data set but annotated with the corresponding failure reason.

## Results

### Evaluation of Template-Based Docking and Free Docking with Alternative Pose Selection Strategies

#### The Enriched Astex Set

First, a benchmark set for validating free and template-based docking was compiled. The well-established Astex Diverse set served as the basis for this benchmark.^38^ As the Astex Diverse set contains only one protein–ligand complex per target, it was enriched with structures of the same protein target bound to different ligands contained in Binding MOAD, restricting the selection to structures with a resolution ≤ 2.5 Å. Of the 85 protein targets in the Astex set, 72 were represented in Binding MOAD. In one case, P04818, only the PDB structure in the Astex Diverse set was found. This target was therefore unsuitable for cross-docking experiments and was discarded. The remaining 71 targets in the Astex sets for which at least one other ligand-bound crystal structure was found were included in the enriched set, resulting in a total of 2007 protein–ligand complexes (Table S1). Most of the ligands in this set had physicochemical properties in the drug-like range (Figure S1). The number of protein–ligand complexes per target ranged from a minimum of 2 to a maximum of 322, with a median of 12 (Figure S2).

The underlying assumption for template-based docking is that similar ligands have similar binding modes.^8^ To assess whether this was also the case for the complexes contained in the enriched Astex set, the RMSDs of the MCS of all ligand pairs per target were calculated. For about half of the ligand pairs in the enriched-Astex set, an MCS RMSD ≤ 2 Å was obtained (Figure 1). When only pairs of ligands with a Tanimoto similarity ≥ 0.6 or ≥ 0.7 were considered, 78 and 81% of the pairs were found to have similar binding modes, respectively. There were very few pairs for which the MCS RMSD was between 6 and 9 Å, especially if a Tanimoto similarity cutoff was used. Surprisingly, however, 120 ligand pairs (8% of the total) showed an MCS RMSD value ≥ 9 Å despite having a Tanimoto similarity ≥ 0.6. Similarly, 40 pairs (6% of the total) with a Tanimoto similarity ≥ 0.7 had MCS RMSD values ≥ 9 Å (Table S2). These ligand pairs originated from only 5 different targets, with nitric oxide synthase I (UniProt ID P29476) being by far the most prominent one (35 out of 40). After removing this target, 89% of the ligands showed an MCS RMSD ≤ 2 Å for both Tanimoto similarity cutoff values (Figure S3). Even if the number of ligands for this target is considerably high (200), only 13% of the pairs showed this behavior. Therefore, this target was still included in the enriched Astex test set, knowing that it will negatively affect the success rate of molecular docking.

**Figure 1:**
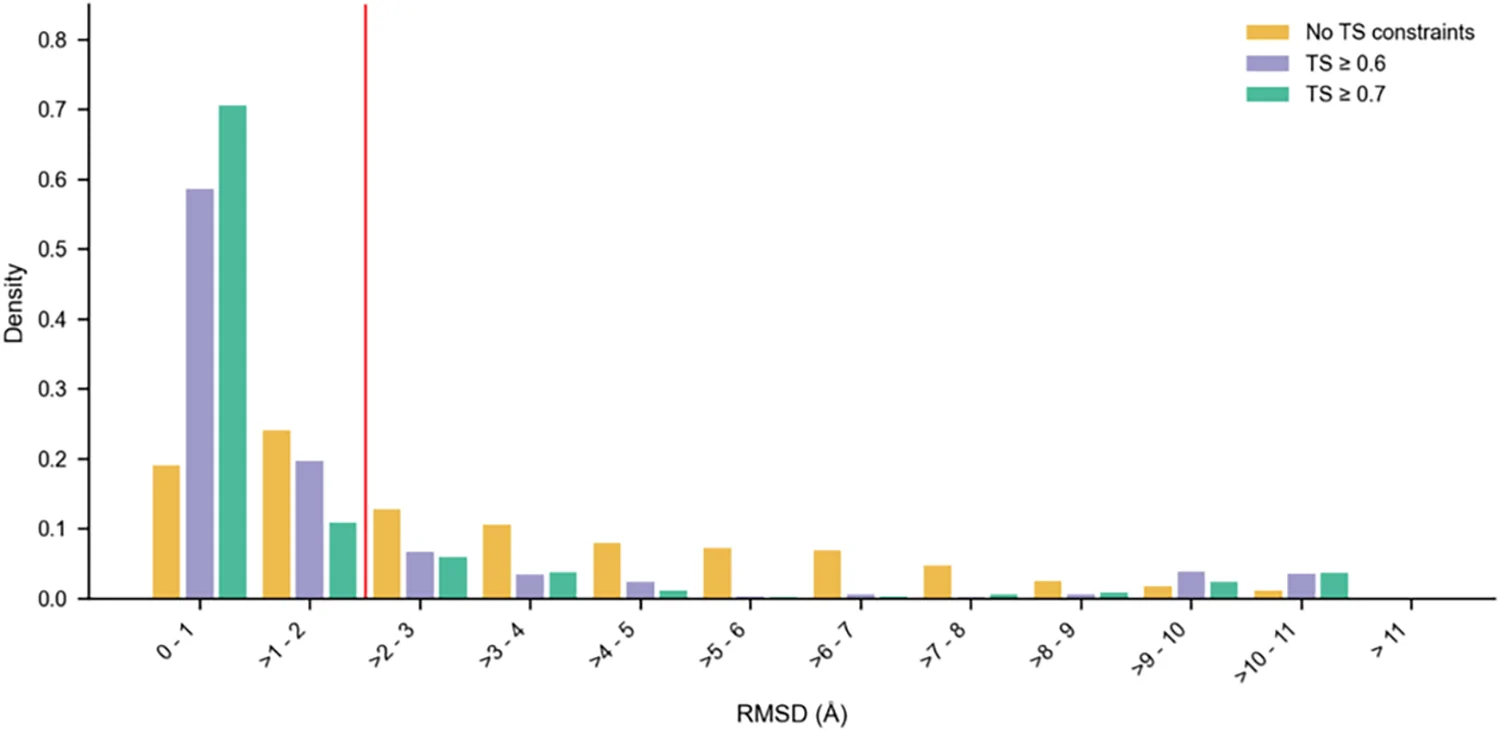
MCS RMSD calculated for all ligands pairs per target in the enriched Astex data set (yellow) and only ligand pairs with a Tanimoto similarity ≥ 0.6 (purple) or ≥ 0.7 (green). The redline indicates the 2 Å cutoff at which poses are generally considered to be close to native.

To further investigate why some ligand pairs had large MCS RMSDs even though they bind to the same targets, pairs with an MCS RMSD ≥ 9 Å were visually inspected. This revealed that in these pairs the ligands had flipped binding modes and the following two common reasons for this were identified: (1) the same functional group attached at both ligand extremities (Figure S4A) or (2) a symmetrical core of the ligand (Figure S4B). However, despite the flipped binding modes, most of the key interactions with the protein were maintained. Overall, these findings confirmed that chemically similar ligands generally adopt comparable binding modes and that the enriched Astex set is suitable for evaluating docking performance, despite a small fraction exhibiting flipped orientations due to molecular symmetry or similar functional groups at opposite ends of the molecules.

#### Cross-Docking Using the Enriched Astex Set

To evaluate different pose generation and filtering strategies, docking experiments were carried out with the enriched Astex set. We focused on cross-docking, i.e. docking of a ligand into a non-native structure of its cognate target (a receptor conformation determined in complex with a different ligand), as it emulates structure-based drug design while providing a ground truth for pose evaluation. Free and template-based docking with or without additional constraints were evaluated. For template-based docking, the ligand contained in the crystal structure used for docking was kept to guide pose generation (template ligand). In the case of free docking, the crystal structure ligand was used as a reference to define the binding pocket but not to guide the docking process (reference ligand). In all experiments, the docking poses were generated using FlexX^45^ and rescored using HYDE.^47^ These methods were chosen because they are well established,^5^ FlexX provides a template-based docking mode, and their combination enables a fast and automated pipeline that can be applied to diverse protein targets and ligand with minimal preprocessing requirements. The RMSD of the resulting poses with the respective crystallographic binding conformation of the same ligand (crystallographic reference) was calculated and poses with an RMSD ≤ 2 Å were considered native-like.

First, for each protein in the enriched Astex set, every ligand was docked into all crystal structures of that protein excluding its native receptor. In this way, all ligands for the same target were paired with all crystal structures of that target and thus implicitly also with all reference/template ligands originally contained in these structures. In the following, we refer to these pairs as “ligand-receptor pairs”. Out of the possible 228,285 ligand–receptor pairs, free docking generated at least one pose for 188,195 of them (Table 2). Template-based docking generated at least one pose for only 155,712 ligand–receptor pairs. The lower number is likely caused by insufficient matches between the template and the ligand to be docked and the reduced search space due to the template constraints. Considering only the top 1 ranked pose for each pair predicted with FlexX, 21% of them were native-like using free docking and 15% using template-based docking (Figure 2, Table 2). Rescoring the poses with HYDE led to improved success rates of 26 and 17%, respectively. When requiring that among the top 10 poses at least one was native-like, the success rate increased to 39% for free docking and 22% for template-based docking using the FlexX scoring function. These values were raised to 41 and 24%, respectively, after rescoring the poses with HYDE.

**Figure 2:**
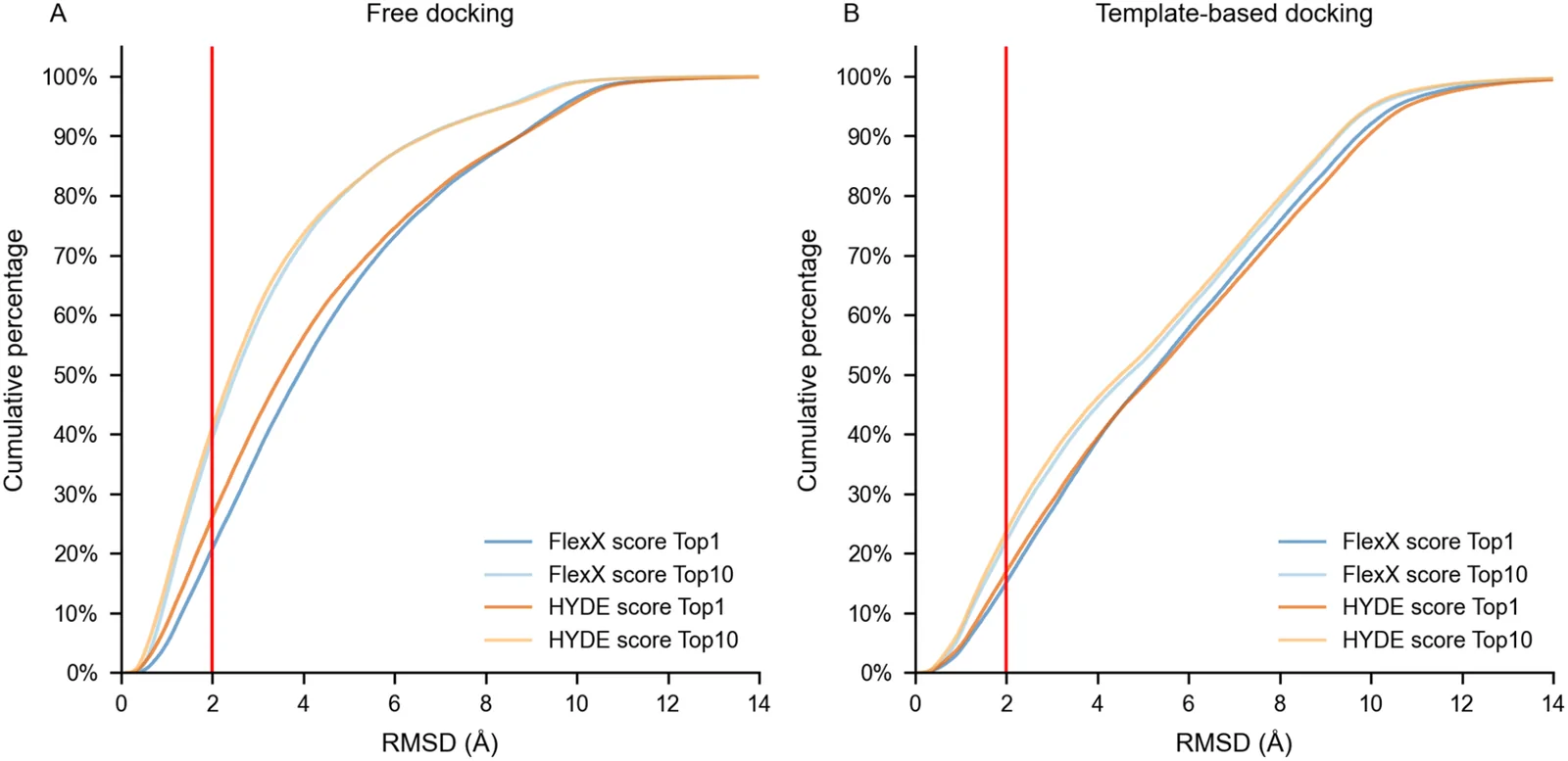
Cross-docking results with pose selection using FlexX score or HYDE score only. The cumulative percentage of the RMSD values relative to the crystallographic binding modes of the docking poses using different selection methods is shown for free docking (A) and template-based docking (B). Dark blue and dark orange: Only the top scoring poses using FlexX score and HYDE score, respectively, were considered. Light blue and light orange: For each receptor–ligand pair, among the top generated 10 poses only the pose with the smallest RMSD value compared to the crystallographic pose was considered. Poses with an RMSD ≤ 2 Å (red line) were considered native-like.

**Table 2:** Cross-Docking Results for Free and Template-Based Docking of the Extended Astex Set^a^

	Free docking		Template-based docking	
Ligand–receptor pairs	228,285			
Ligand–receptor pairs with at least one generated pose	188,195		155,712	
	Pairs with RMSD ≤ 2 Å	Success rate	Pairs with RMSD ≤ 2 Å	Success rate
FlexX top 1	38,963	21%	23,515	15%
FlexX top 10	73,842	39%	34,361	22%
HYDE top 1	48,991	26%	26,330	17%
HYDE top 10	77,242	41%	36,734	24%

a Poses were scored using FlexX or HYDE score and either only the top 1 or the top 10 poses were considered. For each case, the number of ligand-receptor pairs for which at least one native-like pose (RMSD ≤ 2 Å to the crystallographic reference) was generated is listed. The success rate represents the percentage of pairs with at least one native-like pose relative to the total number of ligand-receptor pairs for which at least one pose was generated.

Overall, the results show that correct poses are more frequent among the top 10 docking poses than as the top-ranked pose, indicating that relying only on docking scores is suboptimal to identify the correct binding conformation. This effect was more pronounced for free docking than for template-docking which is likely due to the higher diversity of poses generated with the former method. Further, free docking outperformed template-based docking when no similarity constraints were applied to select the docking template. In all cases, the HYDE score slightly outperformed the FlexX score. Therefore, only results obtained with the HYDE score are presented in the following sections.

#### Pose Selection with MCS RMSD and Tanimoto Similarity Constraints

The relatively low success rates obtained above motivated us to explore if information from an experimentally known binding conformation of a related ligand could be utilized to improve pose selection. Specifically, we evaluated the impact of MCS RMSD thresholds, Tanimoto similarity constraints, and combinations thereof on the identification of native-like poses.

First, we investigated whether restricting the pose selection to certain RMSD thresholds between the atom positions of the MCS of the docked ligand and the reference/template ligand led to a better outcome. For this purpose, all ligands were docked and scored, but only the best scoring pose for each ligand–receptor pair meeting the MCS RMSD cutoff was selected. Three MCS RMSD cut-offs were evaluated: ≤ 1.0, ≤ 1.5 or ≤ 2.0 Å. This led to success rates between 35–42% for free docking (Table 3) compared to 26% for unconstrained pose selection using HYDE scoring (Table 2, Figure 3A). For template-based docking, the success rate improved only to 22–25% compared to the 17% baseline success rate reported above (Table 2, Table 3, Figure 3B).

**Figure 3:**
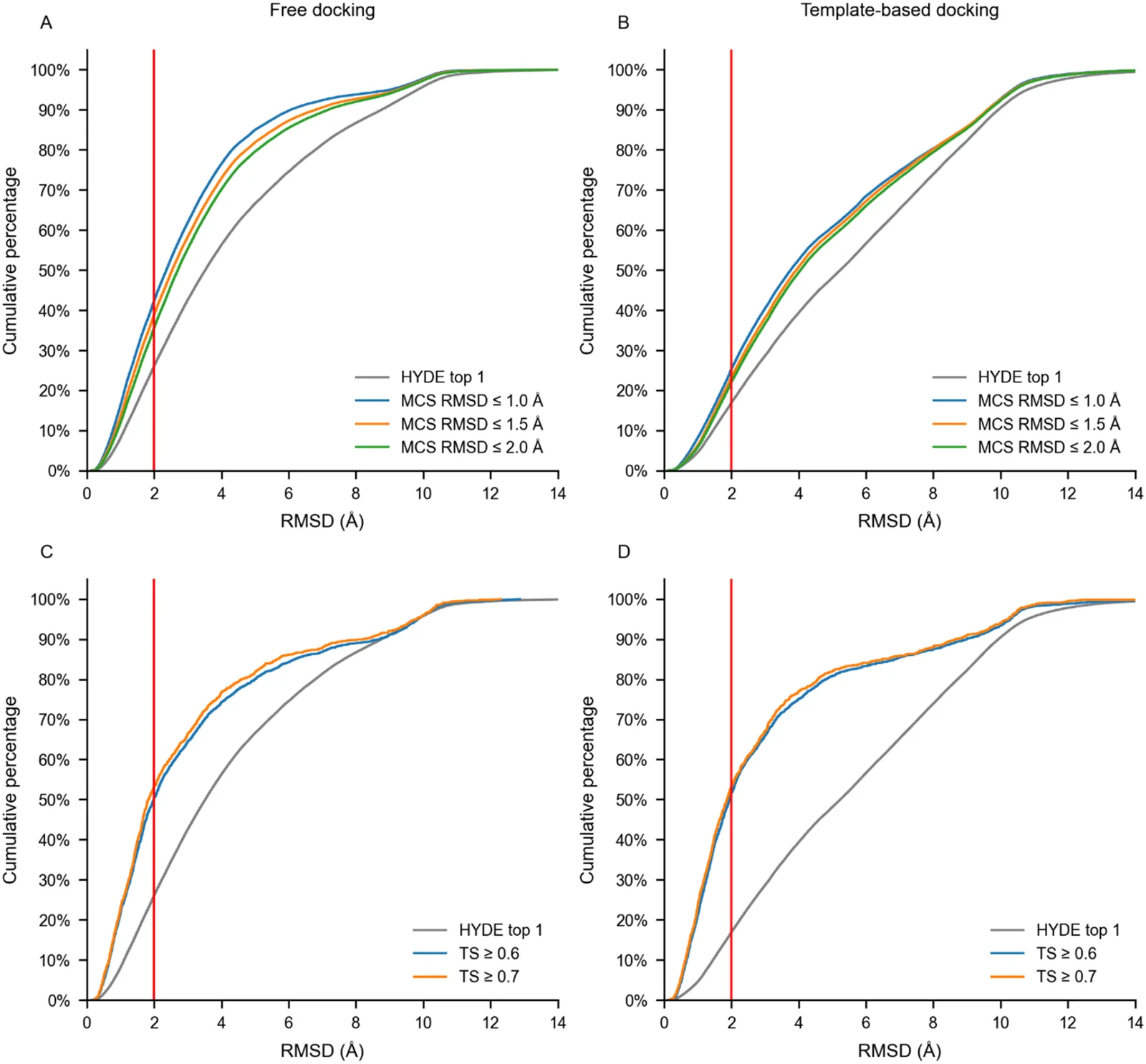
Comparison between cross-docking-based pose selection using HYDE scoring and additional selection criteria. The cumulative percentage is plotted against the RMSD of the docked pose from the native pose. (A, C) Free docking. (B, D) Template-based docking. (A, B) Pose selection based on HYDE score or filtering of the top 10 generated poses based on MCS RMSD cut-offs compared to reference/template ligand. (C, D) Pose selection based on HYDE score using the entire set or only including ligands meeting a Tanimoto similarity (TS) cutoff to the reference/template ligand. In all filtering scenarios, only the highest scoring pose meeting the filtering criteria was kept. Poses with an RMSD ≤ 2 Å (red line) were considered native-like.

**Table 3:** Cross-Docking Results for Free and Template-Based Docking Using either MCS RMSD or Tanimoto Similarity Constraints^a^

	Free docking			Template-based docking		
Ligand–receptor pairs	228,285					
Ligand–receptor pairs with at least one generated pose	188,195			155,712		
	Retained poses (retention rate)		Success rate	Retained poses (retention rate)		Success rate
MCS RMSD ≤ 1.0 Å	60,684	(32%)	42%	57,335	(37%)	25%
MCS RMSD ≤ 1.5 Å	81,777	(43%)	38%	76,506	(49%)	23%
MCS RMSD ≤ 2.0 Å	97,572	(52%)	35%	82,855	(53%)	22%
Tanimoto similarity ≥ 0.6	2,851	(2%)	50%	2,782	(2%)	51%
Tanimoto similarity ≥ 0.7	1,282	(1%)	53%	1,250	(1%)	53%

a The poses were scored using HYDE score and for each ligand-receptor pair, only the best scoring pose meeting the MCS RMSD or Tanimoto similarity cut-off relative to the template/reference ligand was selected. For each filtering strategy, the number of retained poses, the retention rate (relative to the number of ligand/receptor pairs with at least one generated pose) and the success rate are listed. The success rate represents the percentage of selected native-like ligand poses (RMSD ≤ 2 Å) relative to the total number of retained poses using the different filtering criteria.

Subsequently, we applied a Tanimoto similarity constraint between the reference/template ligand contained in the target structure and the ligand being docked to see how their chemical similarity influences docking success. Of the possible 228,354 ligand–receptor pairs (and thus ligand-reference/template combinations), 2,980 had a Tanimoto similarity ≥ 0.6 and 1,316 a Tanimoto similarity ≥ 0.7. Considering only those for docking, with free docking a success rate of 50% was achieved when a Tanimoto similarity cutoff of 0.6 was applied, and 53% when the threshold was raised to 0.7 (Figure 3C, Table 3). The success rates for template-based docking were comparable with 51 and 53%, respectively (Figure 3D, Table 3).

Finally, we investigated the impact of combining MCS RMSD cutoff and Tanimoto similarity constraints on the docking performance (Figure 4, Table 4A). The best success rate (86%) was obtained for free docking when a Tanimoto similarity ≥ 0.7 between the ligand and the reference ligand was required and the RMSD of the MCS of the docked ligand and the reference ligand was < 1.0 Å (Figure 4A, Table 4A). However, a pose was retained for less than half of the ligands contained in the similarity filtered set when using these constraints. Relaxing the Tanimoto similarity and/or the MCS RMSD cutoff led to the inclusion of more poses, but resulted in a drop in the performance (Figure 4C and E, Table 4A). In all investigated scenarios free docking performed better than template-based docking.

**Figure 4:**
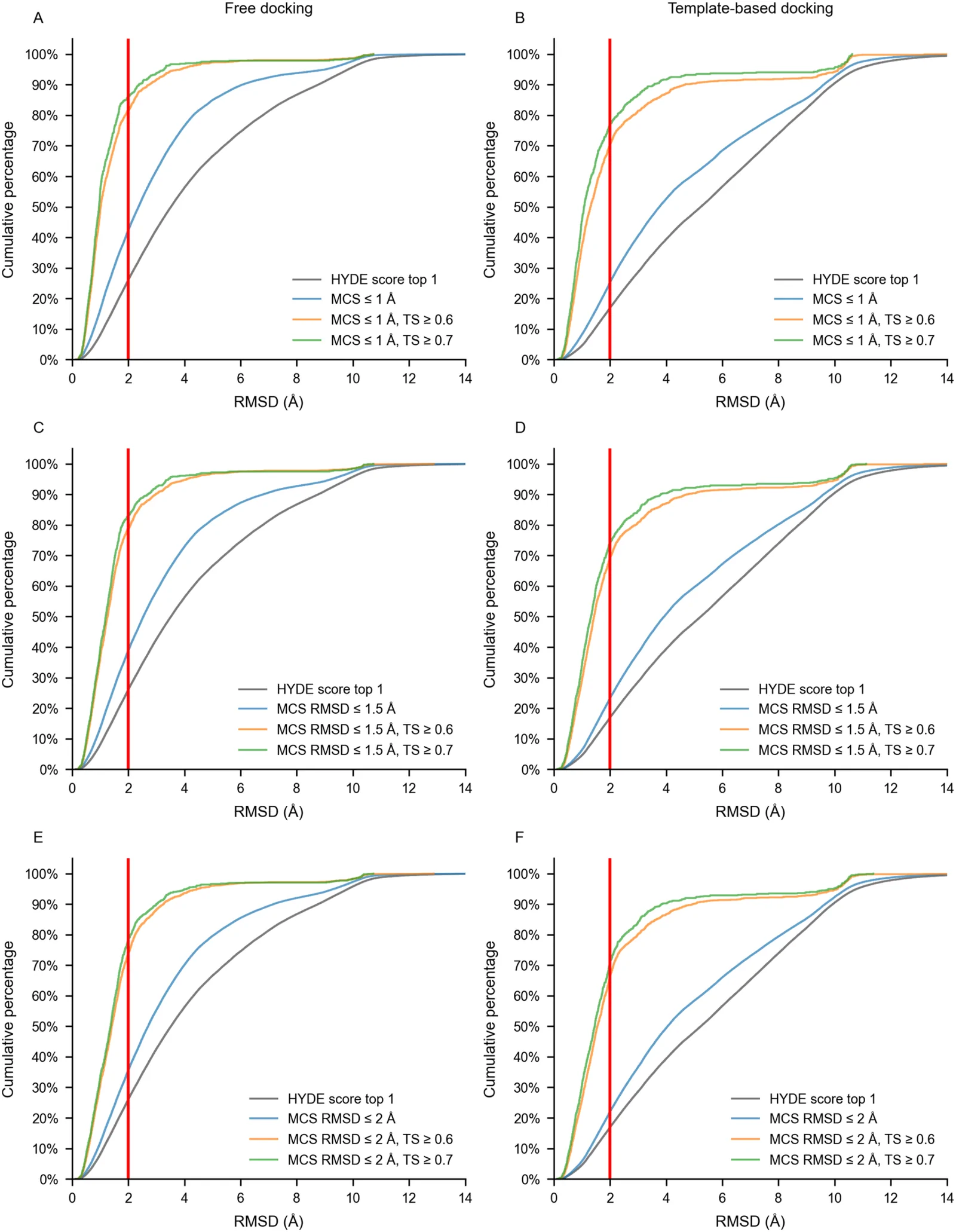
Comparison between cross-docking-based pose selection using HYDE scoring and a combination of different selection criteria. The cumulative percentage is plotted against the RMSD of the docked pose from the native pose. (A, C, E) Free docking. (B, D, F) Template-based docking. Poses were selected out of the top 10 generated poses based on MCS RMSD and Tanimoto similarity constraints. In all filtering scenarios, only the highest scoring pose meeting the filtering criteria was kept. Poses with an RMSD ≤ 2 Å (red line) were considered native-like.

**Table 4:** Cross-Docking Results with Combined Tanimoto Similarity Filtering and MCS RMSD Cut-Offs^a^

Table 4A				
	Tanimoto similarity ≥ 0.6		Tanimoto similarity ≥ 0.7	
Ligand–receptor pairs	2,980		1,316	
	**Free docking**			
	Retained poses (retention rate)	Success rate	Retained poses (retention rate)	Success rate
Ligand–receptor pairs with at least one generated pose	2,851 (95%)	50%	1,282 (97%)	53%
MCS RMSD ≤ 1.0 Å	1,360 (46%)	81%	607 (46%)	86%
MCS RMSD ≤ 1.5 Å	1,752 (59%)	79%	795 (60%)	83%
MCS RMSD ≤ 2.0 Å	2,026 (68%)	74%	916 (69%)	78%
	**Template-based docking**			
	Retained poses (retention rate)	Success rate	Retained poses (retention rate)	Success rate
Ligand–receptor pairs with at least one generated pose	2,782 (93%)	52%	1,250 (94%)	53%
MCS RMSD ≤ 1.0 Å	1,405 (47%)	70%	588 (44%)	77%
MCS RMSD ≤ 1.5 Å	1,708 (57%)	69%	737 (56%)	74%
MCS RMSD ≤ 2.0 Å	1,848 (62%)	66%	799 (60%)	71%

		

Table 4B				
	Tanimoto similarity ≥ 0.6		Tanimoto similarity ≥ 0.7	
Unique ligands	983		674	
	**Free docking**			
	Retained poses (retention rate)	Success rate	Retained poses (retention rate)	Success rate
Unique ligands with at least one generated pose	944 (96%)	50%	648 (96%)	52%
MCS RMSD ≤ 1.0 Å	571 (58%)	82%	361 (54%)	85%
MCS RMSD ≤ 1.5 Å	683 (69%)	78%	439 (65%)	82%
MCS RMSD ≤ 2.0 Å	755 (77%)	73%	499 (74%)	78%
	**Template-based docking**			
	Retained poses (retention rate)	Success rate	Retained poses (retention rate)	Success rate
Unique ligands with at least one generated pose	930 (95%)	56%	633 (94%)	55%
MCS RMSD ≤ 1.0 Å	614 (62%)	76%	373 (55%)	79%
MCS RMSD ≤ 1.5 Å	686 (70%)	74%	434 (64%)	76%
MCS RMSD ≤ 2.0 Å	714 (73%)	72%	458 (68%)	73%

a (A) The poses were scored using HYDE score. For free and template-based docking without additional constraints, only the highest scoring pose was kept. Otherwise for each ligand-receptor pair only the best scoring pose meeting the filtering criteria was retained. The retention rate is the number of retained poses relative to the number of ligand-receptor pairs in the different Tanimoto similarity categories. The success rate is the percentage of poses for which the correct pose (RMSD ≤ 2 Å) was obtained relative to the number of retained poses. (B) As in (A) except that the same ligand for the same target was only considered once. I.e., when the same ligand was docked using different crystal structures of the same target and more than one resulting pose satisfied the selection criteria, only the pose generated using the structure of the reference/template ligand with the highest Tanimoto similarity to the docked ligand was kept.

In the cross-docking experiment described above, if a ligand had several matching reference/template ligands within the applied Tanimoto similarity cutoff, the best pose for each ligand–receptor pair meeting the selection criteria was retained. We therefore further explored how the docking success rates would change if in cases where using several receptors led to acceptable poses, only the pose generated using the structure of the reference/template ligand with the highest Tanimoto similarity to the docked ligand was kept. For free docking, the success rates stayed essentially the same after removing duplicated poses, thus demonstrating robustness of the pose selection pipeline (Table 4B). In contrast, an increase in success rates of up to 6 percentage points was observed for template-based docking. This evidences the premise that template-based docking works best when the ligand and the template ligand are highly similar. When using MCS RMSD constraints after free docking for pose selection, the dependence on ligand similarity is less pronounced.

Finally, we checked how the different pose selection strategies influenced the docking results for the different targets in the enriched Astex set (Figure 5, Figure S5). In general, the success rates improved across the investigated targets for both free docking and template-based docking when Tanimoto similarity and MCS RMSD constraints were applied. For example, using free docking without pose filtering, the median success rate over all targets was only 29% and a success rate ≥ 90% was found for only 4 out of the 70 targets while for 24 targets the success rate was <20%. In contrast, when poses generated with free docking were filtered using an MCS RMSD cutoff of 2 Å and a Tanimoto similarity score ≥ 0.7, the median success rate increased to 97% and a success rate ≥ 90% was achieved for 25 targets, including 19 for which the success rate was 100% In addition, 2 targets had a success rate that ranged from 80 to 90%, and none of the targets in the set had a success rate ≤ 20%. When removing duplicate ligand poses arising from docking the same ligand into different crystal structures of the same target, the success rates were comparable (Figure S6).

**Figure 5:**
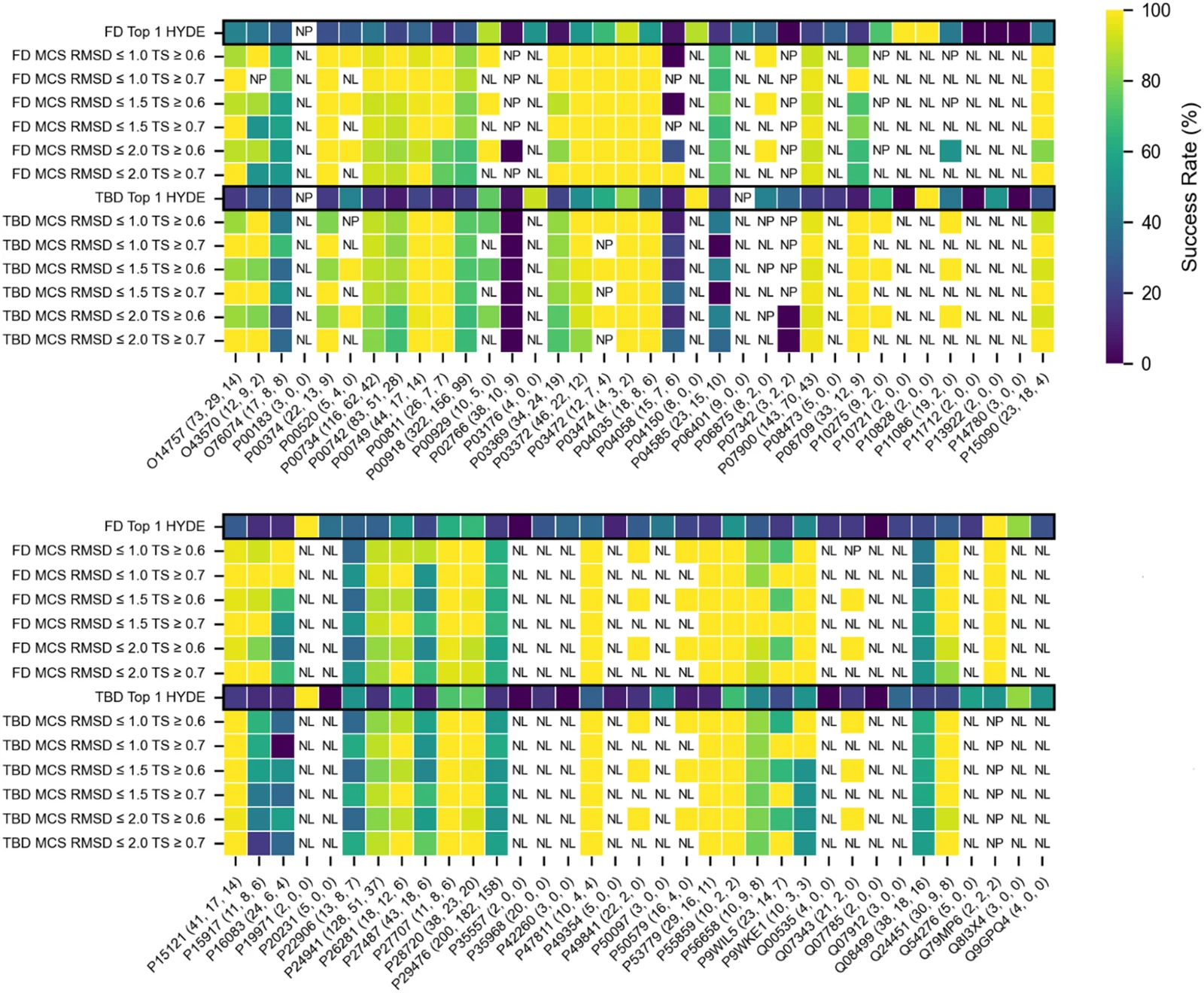
Heatmap showing the success rates by target across different pose selection methods. The *x*-axis lists the UniProt ID of each target, followed by the total number of ligands in parentheses, along with the number of ligands with at least one other ligand with a Tanimoto similarity ≥ 0.6 or ≥ 0.7 within this set. The *y*-axis lists the docking methods and filtering criteria that were applied. The cells are color coded according to the success rate which was calculated as the number of selected poses with an RMSD ≤ 2 Å relative to the number of ligands which met the filtering criteria. The docking results obtained without filtering are marked with a black box. “NP” indicates no poses met the filtering criteria; “NL” denotes no ligands with the specified Tanimoto similarity. FD: free docking; TBD: template-based docking; TS: Tanimoto similarity.

To investigate the reasons for incorrect pose prediction and selection, a more in-depth analysis was conducted on the free docking results with pose filtering based on MCS RMSD cutoff of 2 Å and a Tanimoto similarity score ≥ 0.7. Out of 916 poses selected with this strategy (Table S3A), only 27 poses showed an RMSD ≥ 8 Å to the corresponding crystallographic reference. For the majority of these ligands (21 out 27), the MCS RMSDs calculated with the crystallographic determined pose of the ligand being docked and the crystallographic pose of the reference ligand was ≥ 8 Å as well (Table S3). This shows that the inaccuracies largely derived from differences in the experimentally determined binding conformations for some similar ligands in the enriched Astex diverse set as described above. As an MCS RMSD check of the docked ligand was conducted, for the few cases in which similar ligands adopted different binding modes, inevitable wrong binding poses were enforced using this filtering criteria.

### Compiling a Data Set of High-Quality Modeled Protein Ligand Complexes

Based on the above cross-docking validation of different filtering criteria, we implemented a pipeline to produce a comprehensive data set containing high-quality modeled binding poses for ligands with annotated activity values. This set was termed DockBind. Free docking was selected for our pipeline as it generally performed better in our validation. Further, using a Tanimoto similarity ≥ 0.7 and an MCS RMSD cutoff ≤ 2 Å gave a similar success rate as a Tanimoto similarity ≥ 0.6 and an MCS RMSD cutoff ≤ 1.5 Å (Table 4). However, a larger fraction of the generated poses was retained when applying the former pose filtering criteria. Therefore, Tanimoto similarity ≥ 0.7 and an MCS RMSD ≤ 2 Å cut-offs were included in our pipeline.

#### Enriching Binding MOAD

To compile a comprehensive list of reference ligands to assemble DockBind, the Binding MOAD data set was used as a starting point. Only protein–ligand complexes containing small molecule ligands were selected from Binding MOAD. This resulted in a total of 10,976 protein–ligand complexes, corresponding to 1,281 unique targets. The last release of Binding MOAD dates from 2020, therefore, the data was supplemented with structures from the PDB released between then and June 2024. As a result, 5,800 additional protein–ligand complexes were included. These complexes covered 414 unique targets. Notably, 838 of the selected complexes corresponded to 43 targets that were not previously represented in the Binding MOAD data set, with the most frequent being the SARS-COV-2 replicase (UniProt ID: P0DTD1). Combining the two data sets yielded the enriched Binding MOAD set containing 16,776 reference structures spanning across 1,324 unique protein targets. Most targets had between 1 and 10 ligands, with a maximum of 673 and an average of 13 (Figure S7).

#### Validation of the Docking Pipeline

To evaluate how the docking pipeline described above would perform with targets not contained in our benchmark set, we applied it to the newer PDB structures retrieved to enrich Binding MOAD and that were not contained in the enriched Astex set. Additionally, only reference-ligand pairs having a Tanimoto similarity ≥ 0.7 were retained as this cutoff was used in the docking pipeline. The final validation set included 1,039 unique protein–ligand complexes spanning 121 different targets accounting for 2,830 possible cross-dockings. Although all the eligible cross-dockings were performed, retention and success rates were calculated at the ligand level. When multiple reference structures were available for the same ligand, only one result was retained, selecting the pose obtained with the most similar reference ligand among those satisfying the MCS RMSD criterion. This ensured that each ligand contributed at most once to the analysis. Using the established 2 Å MCS RMSD threshold, an acceptable pose was obtained for 834 cross-docking pairs (80% retention rate) across 106 targets (Table 5). Of these, 675 reproduced the experimental binding mode, corresponding to a success rate of 81%. To estimate confidence intervals (CI), bootstrapping was applied with UniProt targets as the resampling unit. This resulted in a 95% CI of 74–86%. Thus, performance on this independent set was even higher than on the enriched Astex set (Table 4b) giving confidence that with this pipeline high-quality poses can be produced. As seen above, increasing the stringency of the MCS-RMSD cutoff progressively enriched for native-like poses, albeit at the expense of coverage.

**Table 5:** Cross-Docking Results Using Free Docking with Combined Tanimoto Similarity Filtering (≥ 0.7) and MCS RMSD Cut-Offs^a^

	Free docking	
Unique ligands	1,039	
	Retained poses (retention rate)	Success rate (95 % CI)
Unique ligands with at least one generated pose	1,024 (99 %)	54 % (47-62 %)
MCS RMSD ≤ 1.0 Å	665 (64%)	89% (81–93%)
MCS RMSD ≤ 1.5 Å	787 (76%)	84% (76–90%)
MCS RMSD ≤ 2.0 Å	834 (80%)	81% (74–86%)

a The poses were scored using HYDE score. For docking without additional constraints, only the highest scoring pose was kept. Otherwise for each ligand-receptor pair only the best scoring pose meeting the filtering criteria was retained. Each ligand for the same target was only considered once. I.e., when the same ligand was docked using different crystal structures of the same target and more than one resulting pose satisfied the selection criteria, only the pose generated using the structure of the reference ligand with the highest Tanimoto similarity to the docked ligand was kept. The retention rate is the number of retained poses relative to the number of unique ligands. The success rate is the percentage of poses for which the correct pose (RMSD ≤ 2 Å) was obtained relative to the number of retained poses. Confidence intervals (CI) were calculated using bootstrapping with UniProt targets as the resampling unit.

#### Ligand Selection and Pose Generation

Subsequently, ligands with annotated affinity data were selected from ChEMBL and BindingDB based on the Tanimoto similarity to the reference ligands in the enriched Binding MOAD set, and their poses were generated (Figure 6). The binding modes were modeled using free docking and filtered based on MCS RMSD as described above. If multiple poses satisfied the MCS RMSD cutoff, the one with the best HYDE score was kept. Finally, if several possible reference ligands were available and the poses generated using the crystal structures of these ligands passed the MCS RMSD filtering criteria, only the pose generated with the receptor of the reference ligand with the highest Tanimoto similarity was kept.

**Figure 6:**
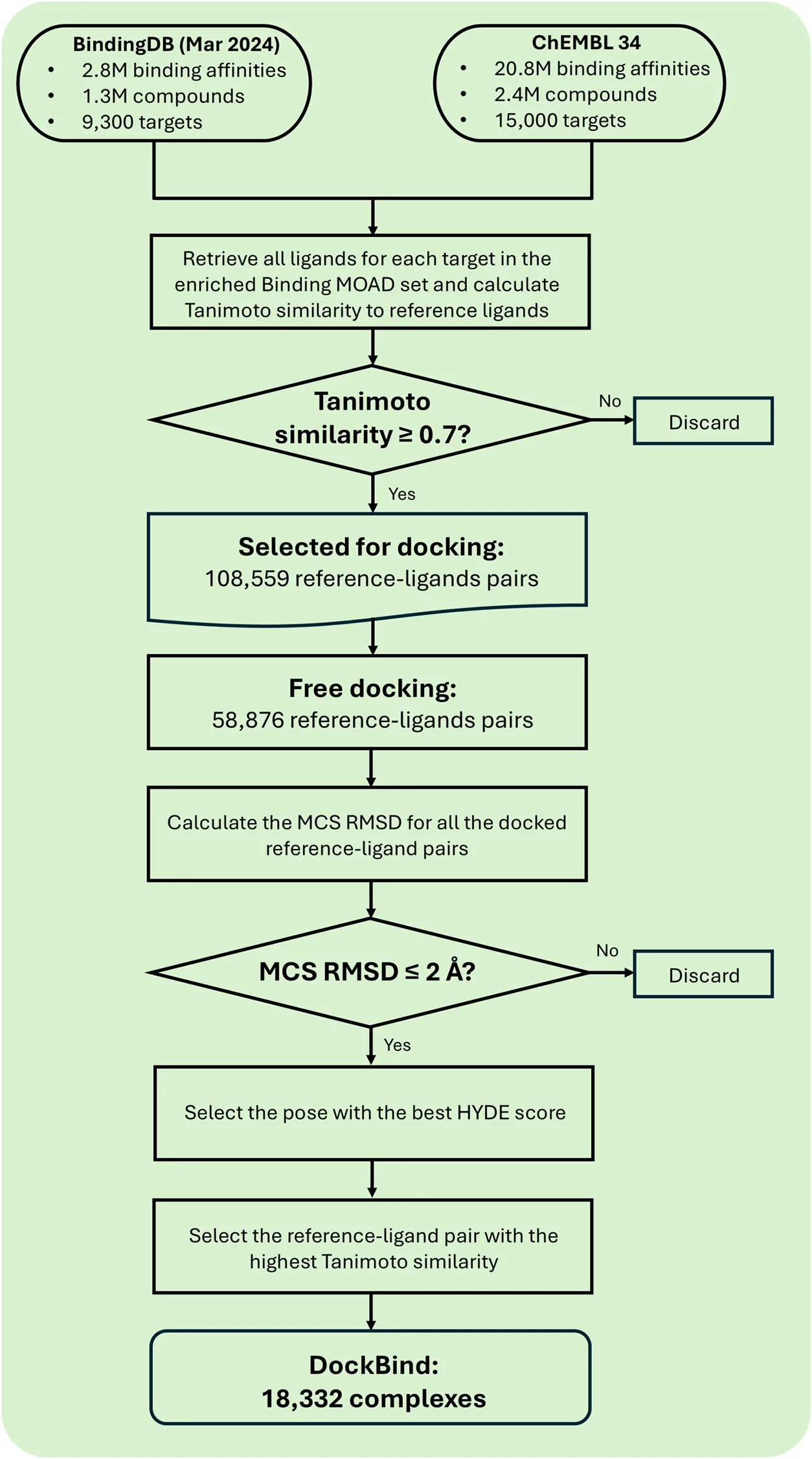
Workflow used to generate the DockBind set. Starting from ChEMBL and BindingDB, ligands for targets in the enriched Binding MOAD data set were selected. Next, ligands with a Tanimoto similarity ≥ 0.7 with at least one reference in the enriched Binding MOAD were retrieved. These ligands were docked with FlexX and poses with an MCS RMSD ≤ 2 Å to the reference were included in the DockBind data set. If multiple poses were available, the one generated with the reference ligand that had the highest Tanimoto similarity was selected.

To enforce nonredundancy in the final DockBind data set, a structure-level harmonization step was performed after docking and pose selection. For each reference ligand, the chemical diversity of the corresponding docked compounds was assessed using the SD file of the docked pose. Identical compounds sharing the same reference ligand were found and grouped. Inspection of the duplication groups revealed multiple sources of redundancy. As expected, one source of redundancy arose from the partial overlap between ChEMBL and BindingDB. Therefore, only one identifier and one associated pose were kept and annotated with the affinity data from ChEMBL as this was considered the more trustworthy source. Further, both in ChEMBL and BindingDB, racemic or stereochemically unspecified entries are stored separately from fully specified isomers. During ligand preparation and conformer generation, unspecified representations will resolve into defined stereochemical structures, leading to duplicate docked objects with distinct database identifiers and potentially different affinity annotations. To address this issue, entries with fewer defined stereocenters as the parent compound were removed in favor of fully specified representations effectively deduplicating them and ensuring pairing of the correct isomer with the related affinity data. A similar issue arose because the same compound could be represented by multiple identifiers corresponding to different salt forms. Following ligand preparation for docking, these salt forms collapsed into a single parent compound, resulting in duplicate entries associated with different identifiers. These redundancies were solved by retaining only the free-form while preserving affinity data from the salt form only if the affinity type was different from the one associated with the free-form entry. Furthermore, genuine internal duplications were observed within BindingDB, where chemically identical compounds were represented by distinct monomer identifiers, occasionally differing in tautomeric or Kekulé representation. In these cases, a single representative pose was retained after manual verification. A conformer generation issue during docking affecting 1,4-disubstituted cyclohexane stereoisomers was causing the last type of duplications. For these compounds, the trans-stereoisomer was systematically produced, even when the cis-stereoisomer were provided, leading to an impossible interconversion and causing duplications. In this case, only the physically consistent stereoisomer was retained. Together, this postdocking structure validation ensured that each protein–ligand complex in DockBind is truly unique and associated with the plausible affinity annotation.

#### The DockBind Data Set

The final DockBind data set contains 18,332 modeled ligand-protein complexes (Figure 6). These are spread across 612 protein targets and were generated using 4,856 reference ligands. Overall, 379 distinct protein families (Pfam) are represented, although their distribution is uneven (Figure S8A). The median number of complexes per Pfam annotation is 10, and 197 annotations (52%) are represented by 10 or fewer complexes. The protein kinase domain (PF00069) is the most represented one, occurring in 3,334 complexes (18% of the data set, (Figure S8B). The five and ten most represented Pfam annotations covered 48 and 61% of DockBind complexes, respectively, while occurring across 26 and 32% of unique protein targets. The number of docked ligands per target varies between 1 and >100 (Figure 7A). Because the DockBind data set was made by docking ligands similar to reference ligands, structurally related compounds are frequently represented within individual targets. Among the targets containing at least two compounds, 94% have at least one identified chemical series (Table S3A). Across all targets, the median number of series was one and for a typical target, approximately two-thirds of the associated compounds belonged to its largest chemical series. Despite this local enrichment, scaffold diversity across the full data set remained broad. Among the 18,332 complexes 6,899 Bemis-Murcko scaffolds can be found, with the ten most represented scaffolds accounting for only 5.4% of the DockBind (Table S3B). A total of 19,677 activity values are associated with the protein–ligand complexes – 63% retrieved from ChEMBL and the remaining 37% from BindingDB. The majority of the affinity data in DockBind are IC_50_ values (72%), followed by *K*_i_ (20%) and *K*_d_ values (8%).

**Figure 7:**
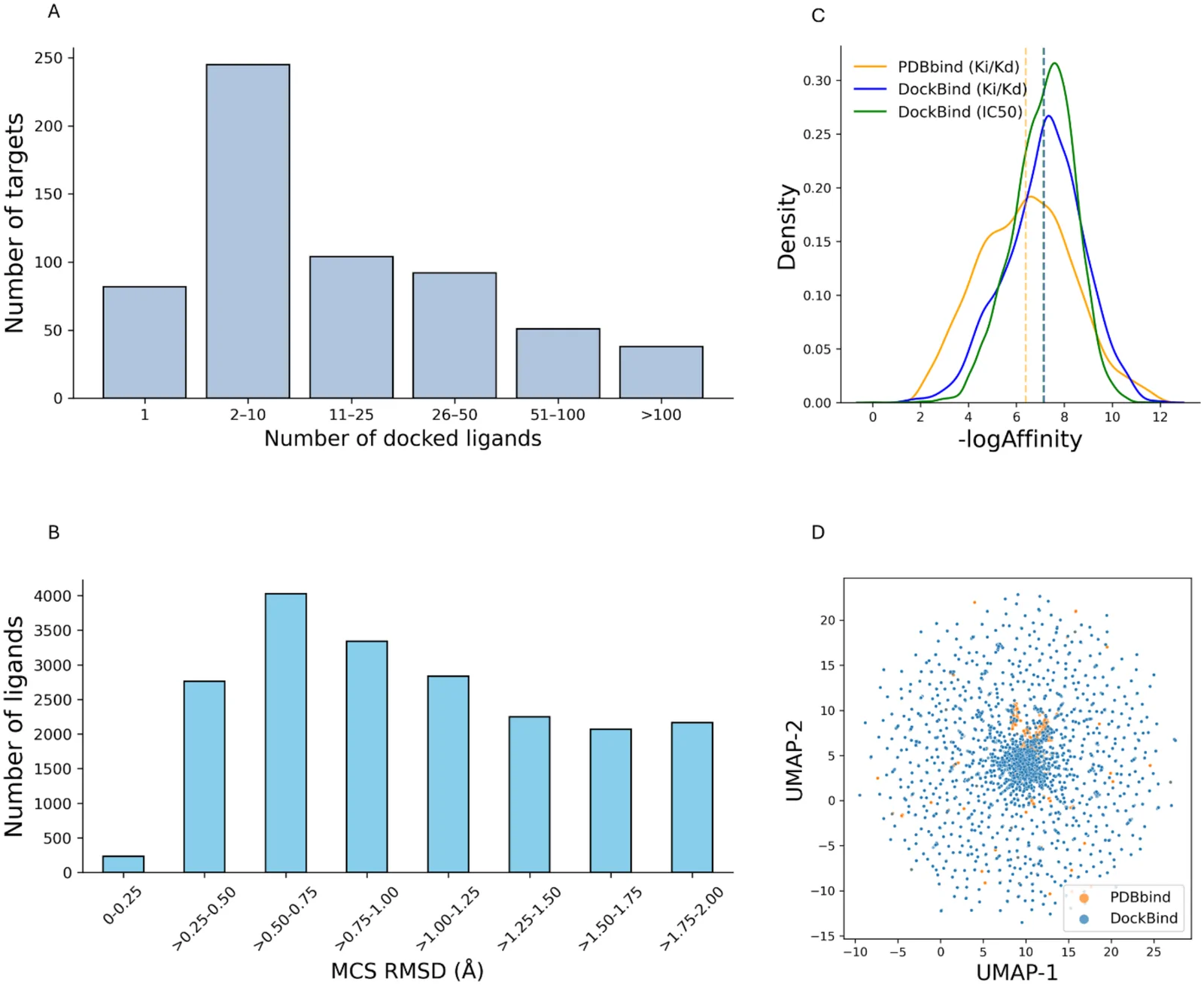
Overview of DockBind data set. (A) Distribution of the number of docked ligands per target. (B) Distribution of the MCS RMSD calculated between the docked poses and the reference ligands. (C) Distribution of the log-transformed binding affinities in DockBind and PDBBind. The dashed lines represent the mean values for each set. (D) Chemical diversity visualization using molecular fingerprint-based dimensionality reduction of ligands with annotated *K*_d_ and *K*_I_ values in DockBind (blue) and PDBBind (orange).

To get an indication of the quality of the generated poses in DockBind, the distribution of the MCS RMSD values between the docked and reference ligands and the Tanimoto similarity of these pairs was analyzed (Figure 7B). This revealed that over half of the poses (52%) have an MCS RMSD within 1 Å, the threshold for which a success rate of 86% was achieved for the enriched Astex set (Table 4). The quality of the poses was also checked with PoseBusters and, as expected, only very few (2.0%) were flagged, mainly due to steric clashes-related warnings.

Given that the PDBbind v2020 refined set is a commonly used data set for training machine learning scoring functions, we use it as a reference to evaluate the binding affinity distribution and the chemical space coverage of DockBind.^32^ The PDBbind refined set is composed of 5,316 protein–ligand complexes. However, 11 of these ligands could not be parsed by RDKit and were therefore removed. As the PDBbind refined set is annotated only with *K*_d_ and *K*_i_ affinity types, the DockBind data set was split into two subsets. One subset, used for the comparison with PDBbind, contained only *K*_d_ and *K*_i_, while the other consisted exclusively of IC_50_ measurements. The distribution of −log transformed binding affinities revealed notable differences in ligand potency between the DockBind and PDBbind data sets. While the PDBbind distribution is broader and skewed toward lower values with a mean value of 6.4, binding affinities in the DockBind subset combining −log *K*_i_ and −log *K*_d_ is centered toward more potent binders, with a mean value of 7.2 ± 1.6 (Figure 7C). Similarly, the DockBind −log IC_50_ binding affinity distribution is tightly centered around a mean value of 7.1 ± 1.3.

Our analysis of the chemical space covered revealed an increase in chemical diversity for the ligands in DockBind compared to the ligands in PDBbind (Figure 7D). The DockBind data set covers most of the PDBbind chemical space, although small clusters in PDBbind are not represented in DockBind. Furthermore, a dense central cluster of similar ligands was identified for both DockBind and PDBbind, where most compounds share similar chemical properties. However, the peripheral region surrounding this central group of ligands is more densely populated in DockBind than in PDBbind, revealing an increase in the chemical diversity arising from the inclusion of ligands from ChEMBL and BindingDB.

## Discussion

Substantial research effort is currently focused on leveraging AI-based approaches for the development of docking tools and scoring functions. These data-driven approaches often rely on experimentally determined protein–ligand complexes as training and validation data with PDBbind being by far the most used data set for this purpose.^17,21^ However, concerns have been raised in the last years regarding the presence of biases in these data sets and data leakage issues, which hinder generalizability of the trained models and limits performance in real-world drug discovery applications.^20,54–56^ In addition, the availability of experimentally determined protein–ligand complexes is limited by experimental bottlenecks, while binding affinity measurements are widely available on databases such as ChEMBL and BindingDB. In recent years, several data sets have been generated through molecular modeling approaches aiming at closing the gap between structural and activity data.^9,25,35,57,58^ However, a widely adopted strategy focuses on size rather than quality and reliability of the generated conformations and binding affinity data. Therefore, data sets containing computationally generated, high-quality protein–ligand complexes and associated affinity are still required to allow more realistic benchmarking. In turn, this necessitates a docking protocol resulting in close to native docking poses. To create such a protocol, we have carried out a detailed assessment of free and template-based docking using different pose filtering criteria. The setup giving a good balance between the number of generated poses and their quality was then used to compile a data set of high-quality docking poses which can serve as a training and benchmarking set for docking methods and scoring functions.

To benchmark free and template-based docking, the Astex Diverse set was selected as basis. This choice was taken because this set is a diverse collection of protein targets with almost no global sequence similarity among each other, thus providing a broad framework for evaluating docking performance.^38^ In addition, this benchmark set is well established and has been used in many studies. While newer benchmarks such as the CASF-2016 set provide standardized protocols for assessing different aspects of docking and scoring, their design is primarily geared toward scoring-function evaluation and includes multiple complexes for a more limited number of protein targets and ligands.^59^ The Astex Diverse set was enriched with additional structures from Binding MOAD^33^ to increase the availability of experimentally determined ligand-bound structures for each target, thereby providing the structural diversity required to investigate free and template-based docking. This enrichment, however, inevitably resulted in an uneven representation of protein targets, as the number of available structures varies substantially across the PDB and consequently in Binding MOAD (Table S1, Figure S2). This imbalance reflects the underlying availability of experimental structural data, where certain protein targets have been extensively crystallized due to their biological and pharmaceutical relevance, whereas others have limited structural coverage. The enriched Astex set was consequently not intended to provide a uniformly sampled collection of protein targets, but rather to exploit the experimentally available structural diversity to investigate the potential benefits and limitations of different docking protocols.

Validation based on the enriched Astex set showed, maybe surprisingly, that free docking outperformed template-based docking in all but one investigated cross-docking scenarios (Tables 2, 3, and 4). If only the most similar template or reference ligand was used during docking, template-based docking performed best (Figure 4B). In all other cases, free docking performed better. The difference was most pronounced when the lower Tanimoto similarity cutoff to the template ligand was applied. It appears that when allowing for larger template dissimilarity, the native poses of the template and docked ligand may also substantially differ, and template-based docking will then strongly guide the docking toward a faulty pose. Further, the validation highlighted that ligand selection combined with postdocking filtering are critical factors for achieving high-quality binding poses (Figure 4, Table 4). Selecting reference-ligand pairs based on Tanimoto similarity and applying MCS RMSD filtering of the generated poses substantially increases the likelihood of obtaining native-like conformations (Table 4). Although the introduction of these constraints inevitably reduces the number of retained poses, the resulting conformations are of higher quality. Overall, the results of our benchmark with the extended Astex set are in line with the general assumption that similar ligands have similar binding modes (Table 4). However, even if rare, deviations from this behavior were observed, particularly for symmetric ligands or compounds with interchangeable functional groups, where multiple binding orientations are possible. By forcing such ligands to adopt a pose that resembles the template/reference, a wrong binding pose is unavoidably obtained. This disadvantage is clearly outweighed by the much better general performance.

The emergence of free docking with subsequent filtering based on Tanimoto similarity and MCS RMSD cutoff as the winning strategy from our benchmark has an interesting implication: although the information from binding poses from similar ligands is clearly advantageous in selecting the correct poses, it is most beneficial when applied as a second, filtering step. In the initial pose generation, the flexibility of free docking provides an advantage by generating a varied pool of pose candidates. This outweighs the benefit of directly using the template information in template-based docking which leads to more limited variety in poses and consequently, less advantage to be gained from the MCS RMSD filtering. That said, by only using templates that are highly similar to the ligand being docked, the template-based docking performance can be significantly improved and brought to the level of free docking when no other filtering criteria are considered (Table 4B). While we have here investigated only one docking engine-scoring function combination (FlexX and HYDE), we anticipate that the general results and our approach to be applicable to other methods even so optimal cut-offs may vary. In particular, a poor template choice enforcing a wrong binding mode is a general feature of template-based docking whereas the weighing of the template constraints relative to other scoring components will affect the specific optimal similarity cut-offs.

Using the benchmarked pose generation and filtering strategy, we proceeded to assemble a data set of poses associated with affinity data. To achieve a good balance between the quality of the poses, retention of poses after filtering, and total number of generated complexes, we settled on using free docking with filtering based on a Tanimoto similarity ≥ 0.7 and MCS RMSD ≤ 2 Å to the reference ligand (Figure 6). We demonstrated that with this pipeline a good performance on a data set in dependent on the extended Astex set could be achieved (Table 5). Therefore, it is reasonable to expected that although other poses generated with this pipeline are generally of high quality. The resulting DockBind set contains 18,332 complexes. Importantly, over half of the poses (52%) have an MCS RMSD ≤ 1 Å relative to the reference and 78% have an MCS RMSD ≤ 1.5 Å (Figure 7B), the thresholds for which success rates of 85 and 82%, respectively, were achieved for the enriched Astex set (Table 4B). This suggests that a large majority of the poses in DockBind are likely close to native. Importantly, this data set extends the chemical space beyond what is covered by commonly used benchmarks such as PDBbind through the integration of ligands from ChEMBL and BindingDB, reducing analogue bias through the coverage of many different Bemis-Murcko scaffolds (Figure S9B) and improving the representation of less populated regions (Figure 7D).

When augmenting experimental data with computational data, a balance has to be found between the number of computed data points generated and their reliability. For DockBind, we have settled on a rather conservative approach based on a linear assembly pipeline and stringent cut-offs, keeping the receptor rigid for docking and only including affinity measurements arising from direct interactions between the ligand and the target (IC_50_, *K*_d_, *K*_i_). Compared to some other approaches, this has resulted in a smaller set, albeit with a high degree of confidence in the binding poses. While with more complex modeling strategies larger sets can be obtained, the certainty of the modeled poses is often lower. For example, BindingNet v2 contains 689,796 modeled protein–ligand binding complexes. However, according to the authors, only 34% of the structures have a high confidence binding mode; in the authors’ benchmark, this category achieved a success rate of 74%. Furthermore, when modeling poses some flexibility of the binding site residues was allowed, but no validation of the obtained binding-site conformations was presented. Therefore, the true reliability of the modeled poses may be lower than suggested by the reported confidence estimates. In addition, this data set includes EC_50_ measurements, which are typically derived from functional or cellular assays and consequently reflect factors beyond direct protein–ligand binding. Such values introduce an additional layer of uncertainty and are not always directly comparable with other measurements, potentially adding further heterogeneity to the underlying affinity data. Likewise, the recently released MotifLeadDB contains 342,489 models of protein–ligand complexes generated with a proprietary pipeline.^57^ All models were assigned a confidence score, but the relationship between this score and pose accuracy was not benchmarked. Consequently, the reliability of the poses in this data set remains unclear.

DockBind provides a valuable resource for training and benchmarking docking methods and scoring functions. However, like many of its counterparts, DockBind is not free of bias. While DockBind covers a wide range of chemical structures and Bemis-Murcko scaffolds (Figure S9B), protein families are unevenly distributed (Figure S8). As expected based on general research efforts, protein kinases are overrepresented compared to other protein families. Likewise, the affinities of the ligands in DockBind are not equally distributed but follow a bell shaped distribution with mean p*K*_i_/p*K*_d_ and pIC_50_ values of 7.2 and 7.1, respectively (Figure 7C). In addition, each target is associated with a variable number of ligands (Figure 7A) and chemical series (Figure S9B). Depending on the use case, it might therefore be advisable to use a more balanced subset of DockBind. Further, in the DockBind data set *K*_i_, *K*_d_, and IC_50_ values are retained separately because they are not always directly interchangeable.^60^ For machine learning applications, users should therefore take care before pooling these values as equivalent affinity measurements.

Finally, we note that while we have made a large effort to ascertain a correct representation of the ligands, removal of duplicates and high quality of the poses and affinity data in DockBind, less attention was paid to the target structures. In particular, targets were grouped by UniProt IDs but it was not verified if they were any mutated residues in the binding pockets in the crystal structures that might influence the binding affinity of the ligands. This provides a potential direction for improvement which should be addressed in future versions of the data set.

## Conclusions

Here, we have introduced a validated framework for the generation of novel protein–ligand complexes with associated binding affinity data, with a particular emphasis on structural reliability. By combining similarity-based structure selection with postdocking filtering validated on an independent benchmark, the DockBind data set provides complexes with a controlled level of quality. Compared to existing large-scale modeled data sets, DockBind was assembled using a more conservative strategy, prioritizing consistency and interpretability over size, but still providing a good coverage of the chemical space. This makes DockBind particularly well suited for training and benchmarking machine learning scoring functions under conditions that closely reflect practical docking workflows. As a first version, there are clear directions to be explored for improvement of pose selection for DockBind. In particular, future work could focus on replacing heuristic selection criteria with data-driven approaches, where multiple structural and chemical features are collectively analyzed to learn optimal rules for ligand selection and pose acceptance. Such an approach could enable a systematic expansion of DockBind while preserving its reliability. Overall, our work provides strategies for how to boost the quality of computationally generated ligand-protein structures. The resulting data set, DockBind, represents a high-quality resource for structure-based drug discovery and machine learning applications.

## Supplementary Material



## Data Availability

An overview of the extended Astex set is contained in Table S1. The entire extended Astex set together with all generated docking poses and scores, the validation set using newer PDB structures, and the curated DockBind set annotated with Pfam membership and PoseBuster violations together with all docking poses that were generated when compiling this set can be found on Zenodo (10.5281/zenodo.22043523. The scripts that have been used in this work can also be found on Zenodo (10.5281/zenodo.20762428. All software that has been used for this work is described in the Methods section.

## Notes

Additional tables and figures as described above (PDF)
