# Supplementary material for: DockBind: A Quality-Controlled Data Set of Modeled Protein–Ligand Complexes Associated with Affinity Data
Source: J Chem Inf Model. 2026 Oct 26;66(21):13938–55. doi: 10.1021/acs.jcim.6c02093 (PMC13753519; doi:10.1021/acs.jcim.6c02093)
Supplement: Supplementary file 1 [file ci6c02093_si_001.pdf]

Supplementary material

DockBind: a quality-controlled dataset of modelled protein–ligand complexes associated with affinity data

Alberto Rovetta[1], Alberto Memè[1], Hanne S. Antila[1,2,3], Ruth Brenk[1,2*]

1. Department of Biomedicine, University of Bergen, Jonas Lies vei 91, 5020 Bergen, Norway

2. Computational Biology Unit, University of Bergen, Thormøhlensgate 55, 5008 Bergen, Norway

3. Fachbereich Physik, Freie Universität Berlin, Arnimallee 14, 14195 Berlin, Germany

* Corresponding author, Email: ruthbrenk@uib.no

Table S1. The Astex Diverse set targets list mapped with the corresponding UniProt accession number and number of ligands in Binding MOAD. Proteins for which less than 2 ligands were found in Binding MOAD were not included in the extended Astex set.

UniProt ID	PDB code	Target Name	# of Ligands in Binding MOAD
O14757	2BR1	Serine/threonine-protein kinase Chk1	73
O43570	1JD0	Carbonic anhydrase 12	12
O76074	1XOZ	cGMP-specific 3',5'-cyclic phosphodiesterase	17
O93077	1YVF	Genome polyprotein	0
P00183	1P2Y	Camphor 5-monooxygenase	3
P00374	1S3V	Dihydrofolate reductase	22
P00520	1OPK	Tyrosine kinase ABL1	5
P00734	1OYT	Prothrombin	116
P00742	1LPZ	Coagulation factor X	83
P00749	1OWE	Urokinase-type plasminogen activator	44
P00811	1L2S	Beta-lactamase	26
P00918	1OQ5	Carbonic anhydrase 2	322
P00929	1K3U	Tryptophan synthase alpha chain	10
P02766	1TZ8	Transthyretin	38
P03176	1OF1	Thymidine kinase (new UniProt P0DTH5 or Q9QNF7)	4
P03369	1KZK	Gag-Pol polyprotein	34
P03372	1SJ0	Estrogen receptor	46
P03472	1L7F	Neuraminidase	12
P03474	1VCJ	Neuraminidase	4
P04035	1HWI	3-hydroxy-3-methylglutaryl-coenzyme A reductase	18
P04058	1GPK	Acetylcholinesterase	15
P04150	1M2Z	Glucocorticoid receptor	8
P04585	1JLA	Gag-Pol polyprotein	23
P04818	1HVY	Thymidylate synthase	1
P05979	1Q4G	Prostaglandin G/H synthase	0
P06401	1SQN	Progesterone receptor	9
P06875	1GM8	Penicillin G acylase	8
P07342	1T9B	Acetolactate synthase catalytic subunit, mitochondrial	3
P07900	2BSM	Heat shock protein HSP 90-alpha	143
P08473	1R1H	Neprilysin	5

UniProt ID	PDB code	Target Name	# of Ligands in Binding MOAD
P08709	1YGC	Coagulation factor VII	33
P08799	1YV3	Myosin-2 heavy chain	0
P0A6I3	1SQ5	Pantothenate kinase	0
P0AGJ9	1X8X	Tyrosine--tRNA ligase	0
P10275	1Z95	Androgen receptor	9
P10721	1T46	Mast/stem cell growth factor receptor Kit	2
P10827	1NAV	Thyroid hormone receptor alpha	0
P10828	1N46	Thyroid hormone receptor beta	2
P11086	1HNN	Phenylethanolamine N-methyltransferase	19
P11473	1S19	Vitamin D3 receptor	0
P11712	1R9O	Cytochrome P450 2C9	2
P12758	1U1C	Uridine phosphorylase	0
P13689	1LRH	Auxin-binding protein 1	0
P13922	1J3J	Bifunctional dihydrofolate reductase-thymidylate synthase	2
P14780	1GKC	Matrix metalloproteinase-9	3
P15090	1TOW	Fatty acid-binding protein, adipocyte	23
P15121	1T40	Aldo-keto reductase family 1 member B1	41
P15917	1YQY	Lethal factor	11
P16083	1SG0	Ribosyldihydronicotinamide dehydrogenase [quinone]	24
P19971	1UOU	Thymidine phosphorylase	2
P20231	2BM2	Tryptase beta-2	5
P22906	1IA1	Dihydrofolate reductase	13
P24941	1KE5	Cyclin-dependent kinase	128
P26281	1HQ2	2-amino-4-hydroxy-6-hydroxymethyl-dihydropteridine pyrophosphokinase	18
P27487	1N1M	Dipeptidyl peptidase	43
P27707	1P62	Deoxycytidine kinase	11
P28720	1N2V	Queuine tRNA-ribosyltransferase	38
P29476	1MMV	Nitric oxide synthase 1	200
P32449	1OF6	Phospho-2-dehydro-3-deoxyheptonate aldolase, tyrosine-inhibited	0
P35557	1V4S	Hexokinase-4	2

UniProt ID	PDB code	Target Name	# of Ligands in Binding MOAD
P35968	1Y6B	Vascular endothelial growth factor receptor 2	20
P42260	1TT1	Glutamate receptor ionotropic, kainate 2	3
P47811	1YWR	Mitogen-activated protein kinase 14	10
P49354	1MZC	Protein farnesyl-transferase / geranyl-geranyl-transferase type-1 subunit alpha	5
P49841	1Q41	Glycogen synthase kinase-3 beta	22
P50097	1MEH	Inosine-5'-monophosphate dehydrogenase	3
P50579	1R58	Methionine aminopeptidase 2	16
P53779	1PMN	Mitogen-activated protein kinase 10	29
P55859	1V48	Purine nucleoside phosphorylase	10
P56658	1UML	Adenosine deaminase	10
P69905	1G9V	Hemoglobin subunit alpha	0
P9WIL5	1N2J	Pantothenate synthetase	23
P9WKE1	1W2G	Thymidylate kinase	10
Q00535	1UNL	Cyclin-dependent kinase 5	4
Q07343	1XM6	3',5'-cyclic-AMP phosphodiesterase 4B	21
Q07785	1V0P	Cyclin-dependent kinase 2 homolog	2
Q07912	1U4D	Activated CDC42 kinase 1	3
Q08499	1XOQ	3',5'-cyclic-AMP phosphodiesterase 4D	38
Q24451	1HWW	Alpha-mannosidase 2	30
Q54276	1W1P	Chitinase	5
Q79MP6	1JJE	Beta-lactamase	2
Q8I3X4	1Q1G	Purine nucleoside phosphorylase	3
Q9BZ11	1R55	Disintegrin and metalloproteinase domain-containing protein 33	0
Q9GPQ4	1HP0	IAG-nucleoside hydrolase	4
Q9R0M5	1IG3	Thiamine pyrophosphokinase 1	0

Table S2. List of ligand pairs in the enriched Astex set with Tanimoto similarity ≥ 0.6 and an MCS RMSD ≥ 9 Å.

UniProt ID	Ligand pair	RMSD	Tanimoto similarity
P29476	3NLV_3XA - 3NLX_3XB	10.436	1.00
P15121	1X97_FIR - 3BCJ_FIS	9.057	1.00
P29476	4CTP_2IK - 4CTQ_S5D	10.188	1.00
P29476	3JWS_JI4 - 3JX3_JI5	10.617	1.00
P29476	3JWV_J14 - 3JX3_JI5	10.416	1.00
P29476	3NNY_59R - 3NNZ_59W	10.360	1.00
P29476	3JWU_J11 - 3JX3_JI5	10.594	1.00
P29476	3NLM_JRR - 3NLK_JSS	10.504	1.00
P29476	4CX6_Q16 - 4LUX_QJ8	10.546	1.00
P03369	2QI4_MZ6 - 2QI6_MZ8	9.819	0.88
P29476	3NLV_3XA - 3NLZ_3XD	10.148	0.84
P29476	3NLV_3XA - 3PNE_8AX	10.203	0.83
P29476	3NLV_3XA - 3NLY_3XC	10.152	0.83
P29476	4CTQ_S5D - 4CTT_S7K	9.962	0.83
P29476	4KCM_1QF - 4KCO_5H4	10.750	0.81
P29476	3NNY_59R - 3NLK_JSS	10.295	0.80
P29476	3NNZ_59W - 3NLM_JRR	10.326	0.80
P29476	3NLV_3XA - 3SVP_JK5	10.425	0.79
P29476	3UFP_H4W - 3NLM_JRR	9.426	0.79
P29476	3UFO_HW4 - 3NLM_JRR	9.433	0.79
P29476	3UFP_H4W - 3UFS_HW6	10.324	0.77
P29476	3UFO_HW4 - 3UFS_HW6	10.531	0.77
P29476	4K5D_1Q6 - 4K5F_Q14	9.345	0.76

UniProt ID	Ligand pair	RMSD	Tanimoto similarity
P29476	4K5E_1Q7 - 4K5F_Q14	9.249	0.76
P29476	3NLV_3XA - 3SVQ_JK4	10.407	0.75
P04585	6E7J_HWY - 6E9A_J0S	9.905	0.75
P29476	3NLV_3XA - 3NLM_JRR	10.532	0.75
P29476	3NLX_3XB - 3NLK_JSS	10.552	0.75
P29476	3UFP_H4W - 3UFW_HW0	10.082	0.75
P29476	3UFW_HW0 - 3UFO_HW4	10.097	0.75
P29476	3JWU_J11 - 3NLM_JRR	10.121	0.74
P29476	3JX3_JI5 - 3NLK_JSS	10.420	0.74
P29476	3JWS_JI4 - 3NLM_JRR	10.206	0.74
P29476	3JWV_J14 - 3NLM_JRR	10.113	0.74
P29476	4EUX_HW1 - 3UFO_HW4	10.080	0.73
P29476	3UFP_H4W - 4EUX_HW1	10.095	0.73
P29476	3PNG_8CX - 3NLK_JSS	10.525	0.72
P04585	4Q1Y_017 - 5DGU_5B7	9.013	0.72
P29476	3NLV_3XA - 3PNF_8BX	10.338	0.71
P00918	1I9L_INV - 1I9Q_IOF	10.820	0.71
P29476	3NLV_3XA - 3PNG_8CX	10.444	0.69
P00918	1G4J_FFB - 1I9Q_IOF	10.714	0.68
P29476	3UFP_H4W - 3UFU_HW8	10.153	0.68
P29476	3UFO_HW4 - 3UFU_HW8	10.167	0.68
P29476	3JX3_JI5 - 3B3P_JI7	9.071	0.68
P29476	3TYL_AXW - 3SVQ_JK4	9.029	0.68
P29476	3UFO_HW4 - 3UFV_HW9	10.202	0.68

UniProt ID	Ligand pair	RMSD	Tanimoto similarity
P29476	3UFP_H4W - 3UFV_HW9	10.236	0.68
P29476	3NLY_3XC - 3NLK_JSS	10.279	0.68
P00918	1I9P_IOE - 1I9Q_IOF	10.564	0.67
P04585	4Q1Y_017 - 5DGW_5B5	9.051	0.67
P29476	3TYL_AXW - 3NLM_JRR	9.011	0.67
P29476	4CTV_7S7 - 4CTU_S42	10.160	0.67
P29476	3NLZ_3XD - 3NLK_JSS	10.232	0.66
P00918	1I9O_IOC - 1I9Q_IOF	10.568	0.66
P00918	1G4O_BSB - 1I9Q_IOF	10.958	0.66
P29476	3NLZ_3XD - 3NLW_3XF	9.105	0.66
P29476	3NLZ_3XD - 3TYN_CXW	9.001	0.66
P29476	4KCM_1QF - 4KCL_H44	12.533	0.66
P29476	3NLX_3XB - 3UFO_HW4	9.429	0.65
P29476	3NLX_3XB - 3UFP_H4W	9.415	0.65
P00918	1G48_F6B - 1I9Q_IOF	10.979	0.65
P03369	2QI3_MZ5 - 2QI6_MZ8	9.120	0.65
P29476	3NLY_3XC - 3NLW_3XF	9.182	0.65
P29476	3UFR_H5W - 3NLM_JRR	9.501	0.65
P29476	3TYM_08R - 3NLZ_3XD	9.050	0.65
P29476	3NLY_3XC - 3TYL_AXW	9.080	0.65
P00918	1G52_F2B - 1I9Q_IOF	10.664	0.64
P00918	1I9M_INW - 1I9Q_IOF	11.143	0.64
P00918	1I9N_IOA - 1I9Q_IOF	10.698	0.64
P29476	3NLX_3XB - 3TYL_AXW	9.049	0.64

UniProt ID	Ligand pair	RMSD	Tanimoto similarity
P29476	3PNG_8CX - 3TYL_AXW	9.007	0.64
P29476	3PNE_8AX - 3NLK_JSS	10.283	0.64
P29476	3SVQ_JK4 - 3NLK_JSS	10.569	0.64
P29476	3B3O_JI3 - 3JX3_JI5	9.141	0.64
P00918	1G45_FSB - 1I9Q_IOF	10.909	0.64
P29476	3NLZ_3XD - 3TYO_DXW	9.029	0.63
P29476	3UFP_H4W - 3UFT_HW7	9.245	0.63
P29476	3UFO_HW4 - 3UFT_HW7	9.284	0.63
P29476	3PNG_8CX - 3UFO_HW4	9.415	0.63
P29476	3UFQ_HW5 - 3NLM_JRR	9.493	0.63
P29476	3PNG_8CX - 3UFP_H4W	9.404	0.63
P29476	3SVP_JK5 - 3NLK_JSS	10.541	0.63
P29476	3TYN_CXW - 3NLM_JRR	9.057	0.62
P29476	3TYO_DXW - 3NLM_JRR	9.085	0.62
P29476	3NLW_3XF - 3SVP_JK5	9.160	0.62
P29476	3NLX_3XB - 3NLW_3XF	9.201	0.62
P29476	3NLW_3XF - 3PNF_8BX	9.193	0.62
P29476	3PNG_8CX - 3UFR_H5W	9.479	0.62
P29476	3PNF_8BX - 3TYN_CXW	9.036	0.62
P29476	3NLX_3XB - 3UFR_H5W	9.491	0.62
P04585	5DGU_5B7 - 6DJ7_G10	9.022	0.62
P29476	3UFP_H4W - 3JX3_JI5	10.392	0.62
P29476	3UFO_HW4 - 3JX3_JI5	10.394	0.62
P29476	3TYM_08R - 3NLM_JRR	9.108	0.61

UniProt ID	Ligand pair	RMSD	Tanimoto similarity
P29476	3TYM_08R - 3NLY_3XC	9.173	0.61
P29476	3NLW_3XF - 3PNE_8AX	9.135	0.61
P29476	3NLW_3XF - 3SVQ_JK4	9.137	0.61
P29476	3TYM_08R - 3PNF_8BX	9.088	0.61
P29476	3UFS_HW6 - 3NLK_JSS	9.680	0.61
P29476	3UFW_HW0 - 3NLK_JSS	9.613	0.61
P29476	3UFW_HW0 - 3UFQ_HW5	9.301	0.61
P03369	3BXR_DRR - 3BXS_DRS	10.868	0.61
P29476	3NLX_3XB - 3UFQ_HW5	9.497	0.60
P29476	3PNF_8BX - 3NLK_JSS	10.441	0.60
P29476	3PNG_8CX - 3UFQ_HW5	9.478	0.60
P03369	2QI5_MZ7 - 2QI6_MZ8	9.241	0.60
P29476	3NLY_3XC - 3TYN_CXW	9.124	0.60
P29476	3NLY_3XC - 3TYO_DXW	9.156	0.60
P29476	3UFR_H5W - 3UFW_HW0	9.449	0.60

Table S3. The list of reference and-docked ligand pairs with a Tanimoto similarity ≥ 0.7 and an RMSD ≥ 8 Å using free docking. HYDE RMSD: the RMSD of the HYDE optimized pose to the crystallographic reference; CRYST RMSD: the MCS RMSD of the crystallographic poses of the two ligands; MCS RMSD: the MCS RMSD of the docked pose and the reference ligand. RMSD values are expressed in Å.

**UniProt ID**	**Reference**	**Docked**	**HYDE RMSD**	**CRYST RMSD**	**MCS RMSD**	**Tanimoto Similarity**
P29476	3JWU_J11	3JX3_JI5	10.361	10.594	1.460	1.00
P29476	3JX3_JI5	3JWV_J14	10.272	10.416	1.705	1.00
P29476	3JX3_JI5	3JWU_J11	10.727	10.594	0.822	1.00
P29476	4LUX_QJ8	4CX6_Q16	10.504	10.546	1.293	1.00
P29476	4CX6_Q16	4LUX_QJ8	10.342	10.546	1.614	1.00
P29476	4CTP_2IK	4CTQ_S5D	9.941	10.188	1.091	1.00
P29476	4CTQ_S5D	4CTP_2IK	10.125	10.188	0.977	1.00
P29476	3NNZ_59W	3NNY_59R	10.107	10.360	0.654	1.00
P29476	4CTT_S7K	4CTQ_S5D	10.007	9.962	1.682	0.83
P29476	3NLK_JSS	3NNY_59R	10.198	10.295	0.592	0.80
P29476	3NNZ_59W	3NLM_JRR	10.317	10.326	0.659	0.80
P29476	3N5Y_XFL	3N5Z_XFM	9.976	0.131	1.393	0.77
P29476	3N5W_XFJ	5FVT_W67	9.251	3.041	1.070	0.76
P29476	4K5F_Q14	4K5D_1Q6	9.316	9.345	0.916	0.76
P29476	4K5F_Q14	4K5E_1Q7	9.059	9.249	1.987	0.76
P29476	3UFO_HW4	3UFW_HW0	10.314	10.097	1.767	0.75
P29476	4CTP_2IK	4CTU_S42	9.394	0.413	1.976	0.74
P29476	4CTU_S42	4CTP_2IK	9.840	0.413	1.737	0.74
P29476	4CTQ_S5D	4CTU_S42	9.844	0.351	0.739	0.74
P29476	3NLM_JRR	3JWU_J11	10.260	10.121	0.409	0.74

UniProt ID	Reference	Docked	HYDE RMSD	CRYST RMSD	MCS RMSD	Tanimoto Similarity
P29476	3NLM_JRR	3JWV_J14	9.798	10.113	1.454	0.74
P29476	3NLK_JSS	3JX3_JI5	10.328	10.420	1.024	0.74
P29476	3JWV_J14	3NLM_JRR	9.160	10.113	0.784	0.74
P29476	3JWU_J11	3NLM_JRR	10.382	10.121	0.797	0.74
P29476	4CTR_S20	4CTU_S42	10.234	1.856	1.929	0.72
P29476	3NLK_JSS	3PNG_8CX	10.383	10.525	0.645	0.72
P04585	5DGU_5B7	4Q1Y_017	9.522	9.013	1.665	0.72

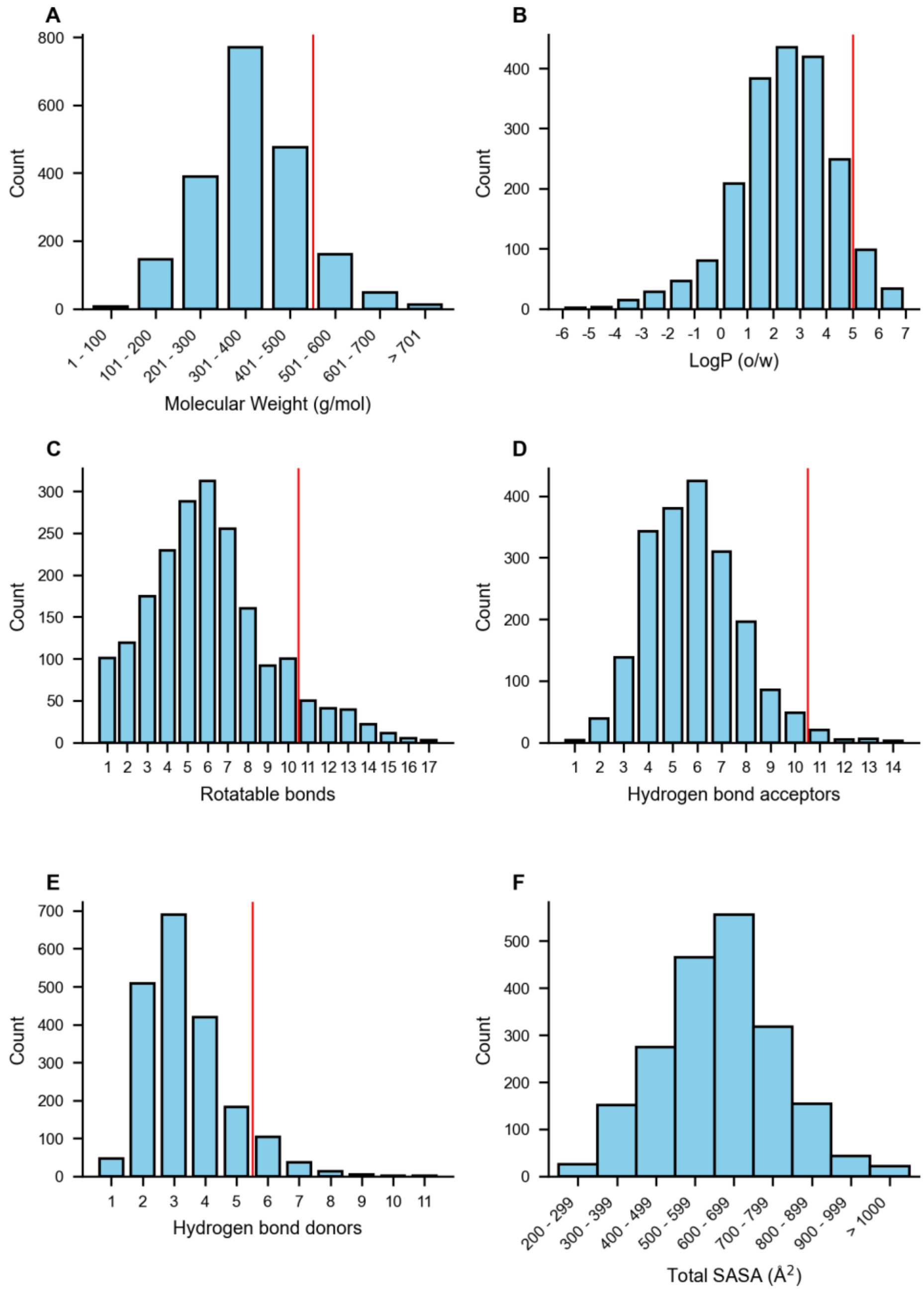

Figure S1. Molecular properties of the ligands in the enriched Astex set. A) Molecular weight (g/mol); B) LogP (o/w); C) Number of rotatable bonds; D) Number of hydrogen bond acceptors; E) Number of hydrogen bond donors; F) Total solvent accessible area (Å^2). Commonly used cut-offs to identify drug-like ligands are marked with a red line.

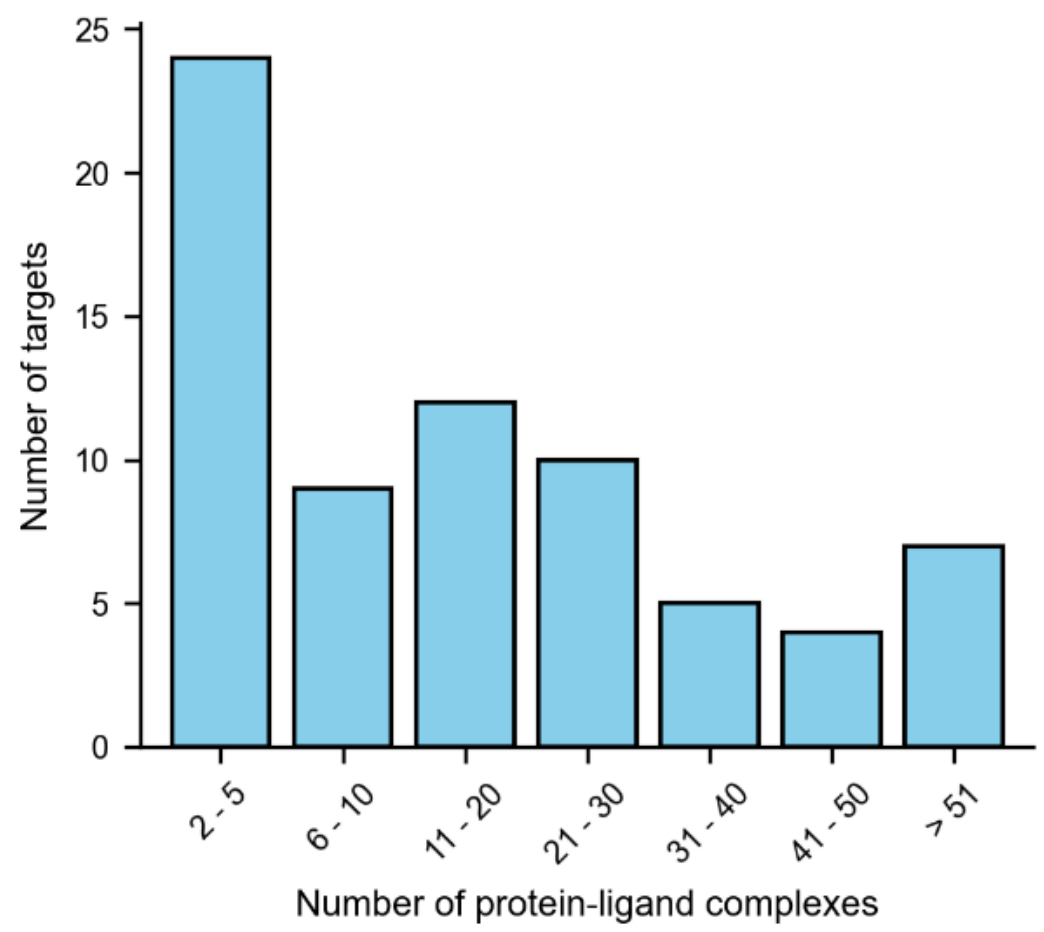

Figure S2. The number of protein-ligand complexes per target in the enriched Astex dataset.

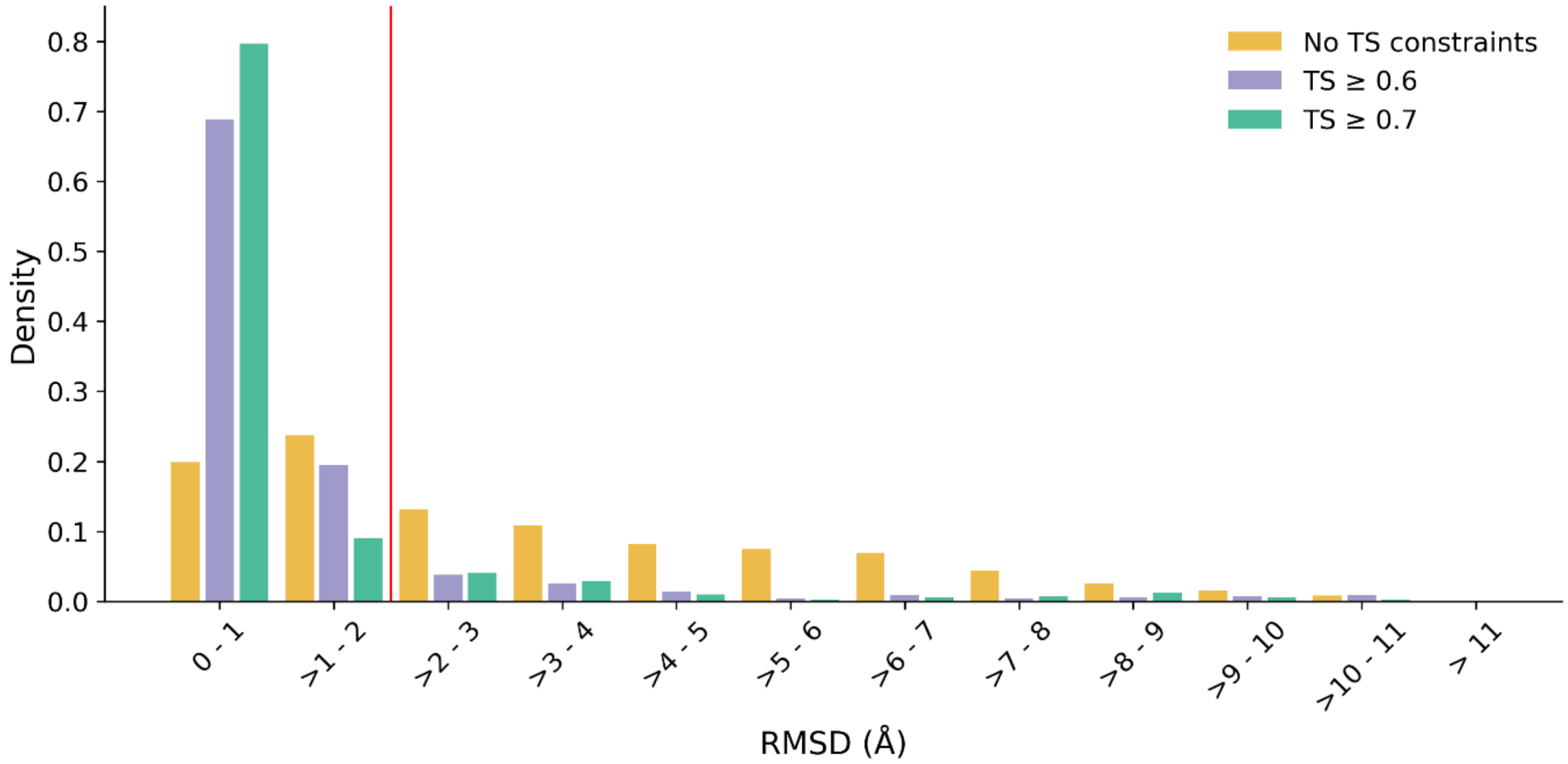

Figure S3. The MCS RMSD calculated for all ligand pairs after removing the target P29476 from the enriched Astex dataset. All ligand pairs are marked in yellow, only ligand pairs with a Tanimoto similarity ≥ 0.6 in purple, and with Tanimoto similarity ≥ 0.7 in green. The red, vertical line indicates the 2 Å cut-off at which poses are generally considered to be close to native.

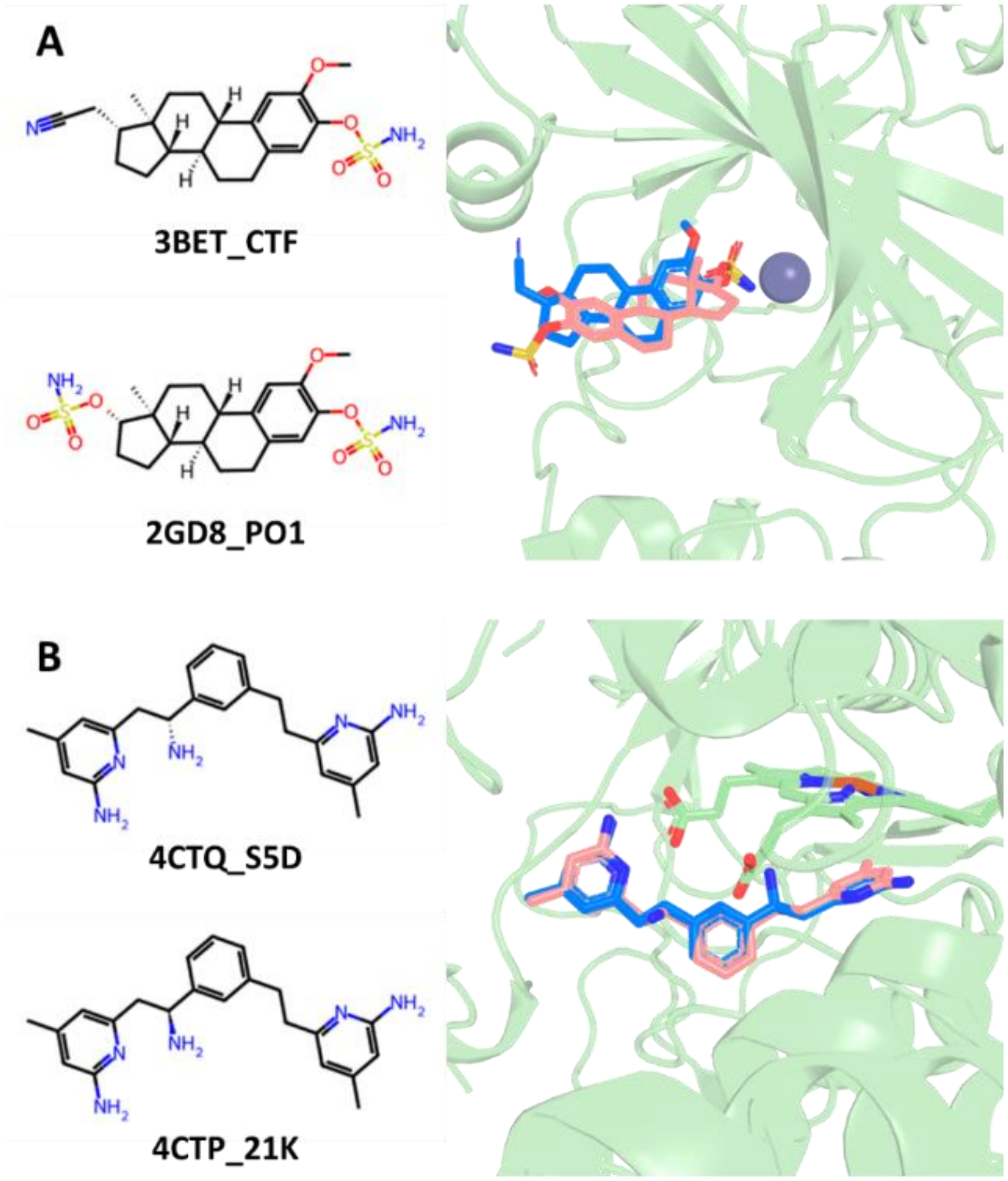

Figure S4. Two examples of ligands with high Tanimoto similarity and high MCS RMSD binding to the same target. A: two carbonic anhydrase II ligands, 3BET_CTF (blue) and 2GD8_PO1 (pink), Zn ion in gray. The ligands have the same functional group (sulfonamide group) required to form interactions with the Zn ion at different ends of the molecule resulting at flipped binding modes. B: Two nitric oxide synthase I ligands, 4CTP_21K (blue) and 4CTQ_S5D (pink). The core of the ligands is identical with the only difference being the stereochemical orientation of the aliphatic amino group resulting in a flipped binding mode forming mostly the same interactions. (Ligand labels include the PDB code and the ligand name (PDB_Ligand).)

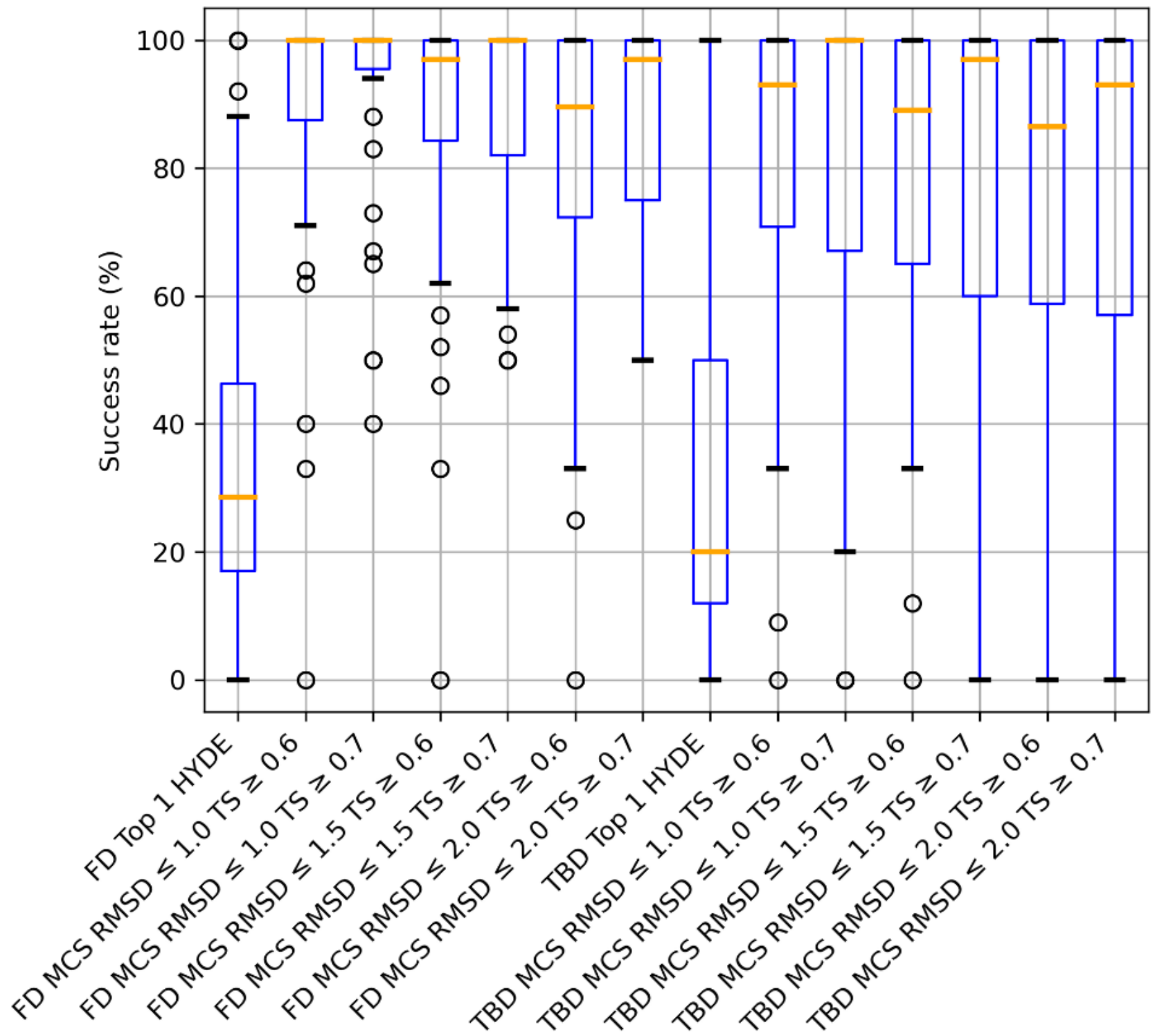

Figure S5. Box plot showing the spread of success rates across different pose selection methods. Targets for which no ligands were left after applying the filtering criteria were ignored. The boxes extend from the first quartile (Q1) to the third quartile (Q3) of the data, with a line at the median. The whiskers extend from the boxes to the farthest data points lying within 1.5x the inter-quartile range (IQR) from the boxes. Outliers are marked with circles and are those past the end of the whiskers. FD: free docking; TBD: template-based docking; TS: Tanimoto similarity.

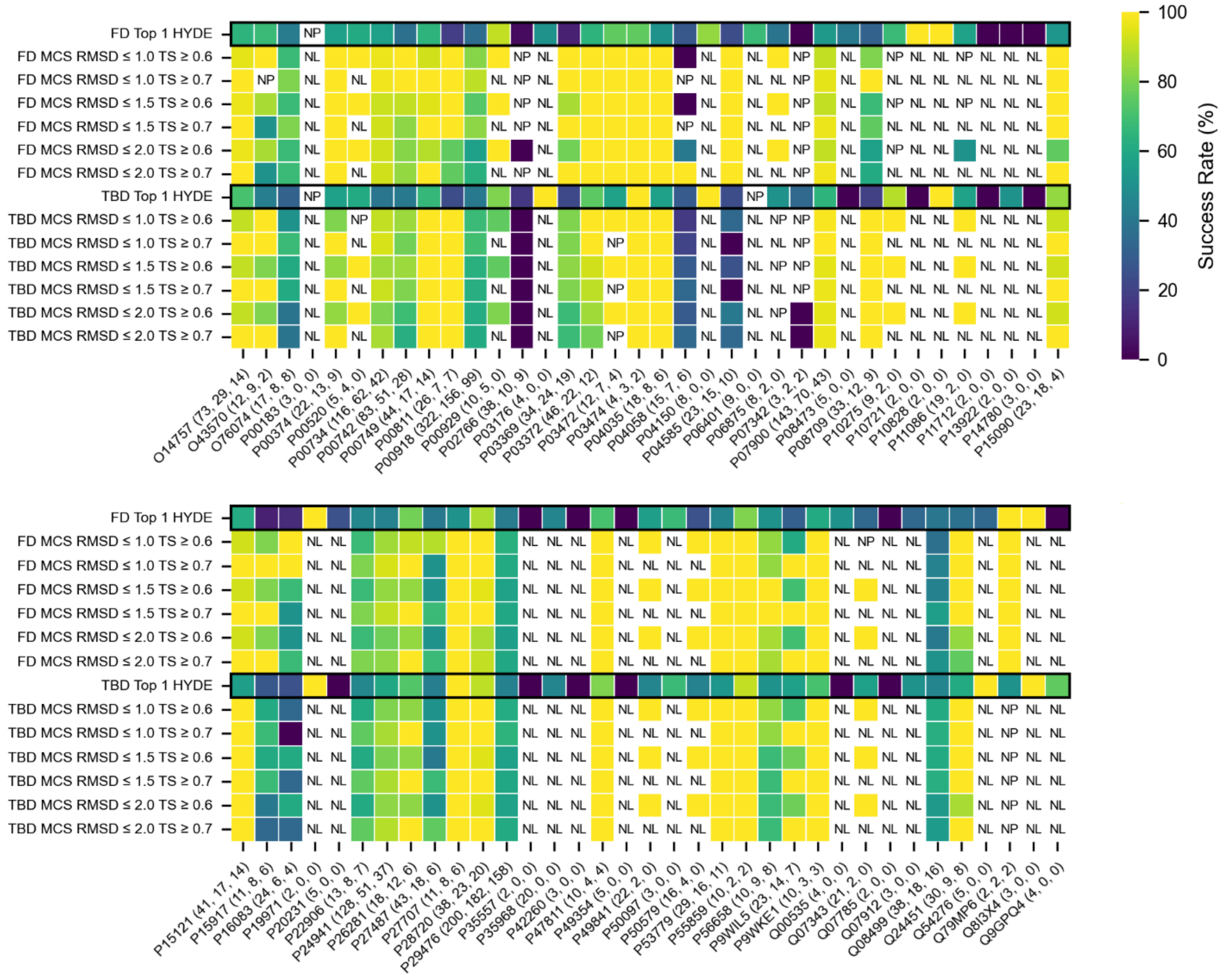

Figure S6. Heatmap showing the success rates by target across different pose selection methods. In cases for which docking poses for the same ligand using different crystal structures of the same protein were obtained meeting the different filter criteria, only the pose generated with the structure which contained the reference/template ligand pair with the highest Tanimoto similarity to the docked ligand was retained. The x-axis lists the UniProt ID of each target, followed by the total number of ligands in parentheses, along with the number of ligands that have at least one other ligand with a Tanimoto similarity ≥ 0.6 or ≥ 0.7 7 within this set. The y-axis lists the docking methods and filtering criteria that were applied. The cells are color coded according to the success rate which was calculated as the number of selected poses with an RMSD ≤ 2 Å relative to the number of ligands met the filtering criteria. The docking results obtained without filtering are marked with a black box. "NP" indicates no poses met the filtering criteria; "NL" denotes no ligands with the specified Tanimoto similarity. FD: free docking; TBD: template-based docking; TS: Tanimoto similarity

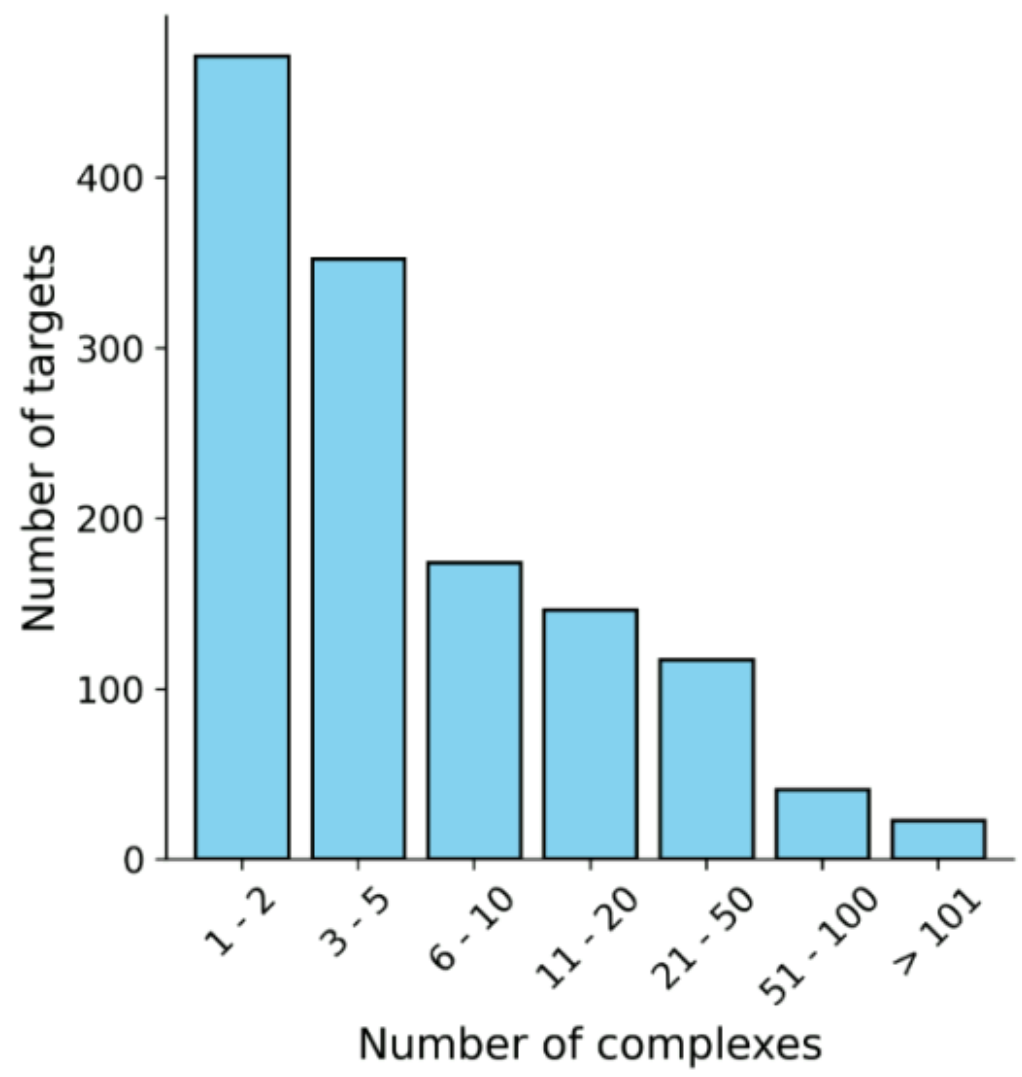

Figure S7. The distribution of the number of protein-ligand complexes per targets in the enriched Binding MOAD dataset.

A

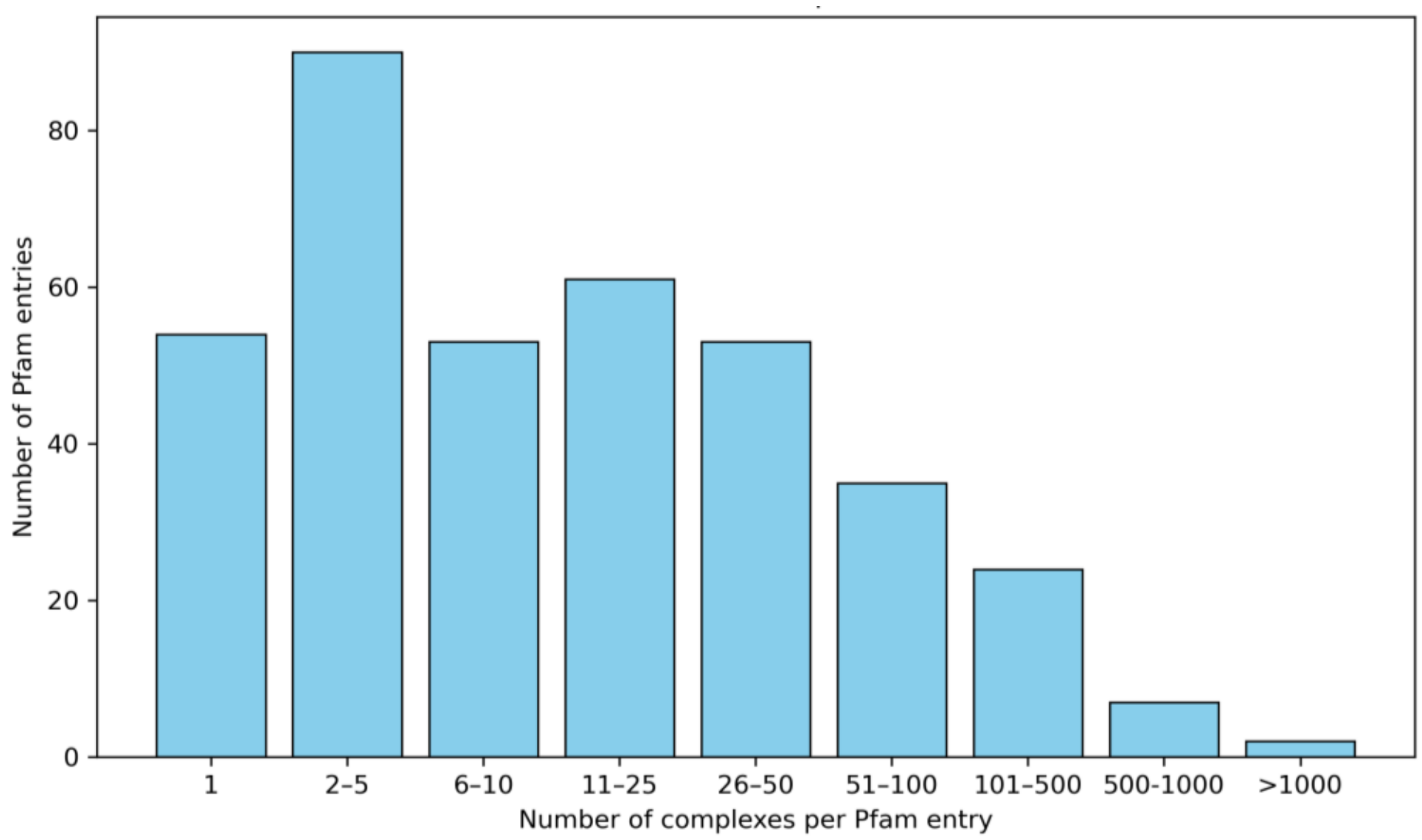

B

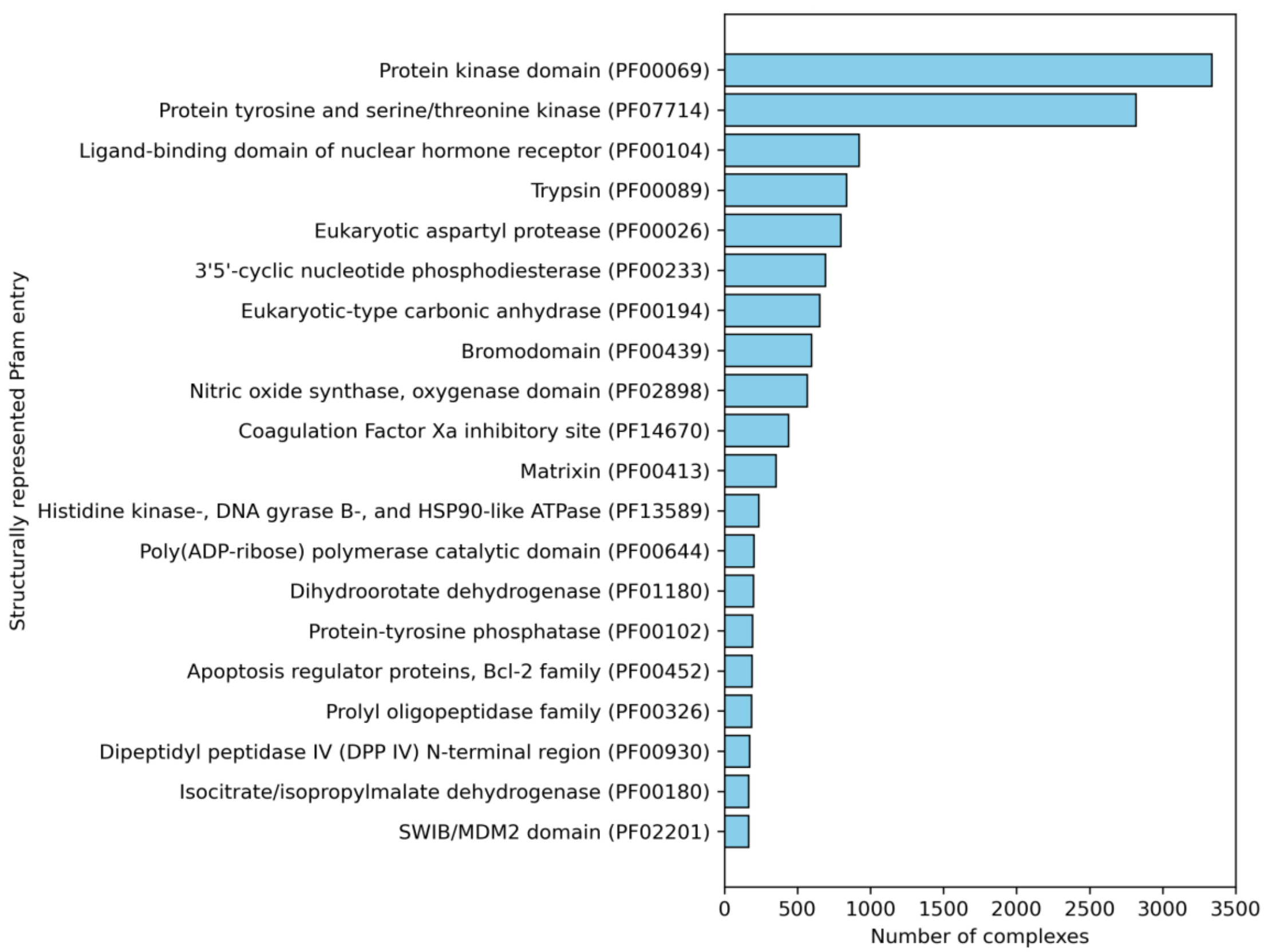

Figure S8. Mapping of protein families (Pfam) to DockBind entries. A: The distribution of the structural Pfam representation in DockBind. B: List of the top 20 most represented Pfam entries in DockBind together with number of complexes per Pfam entry.

A

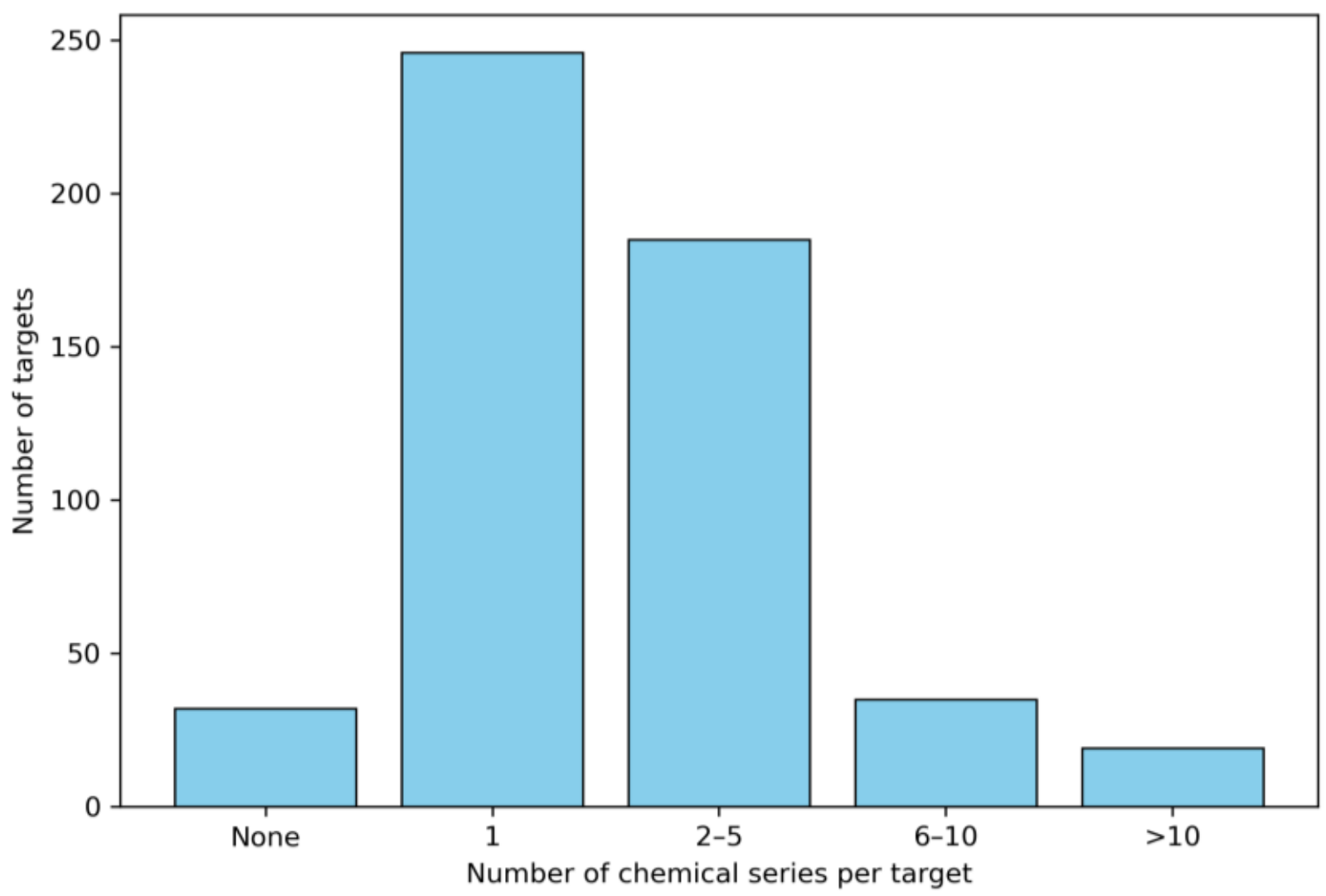

B

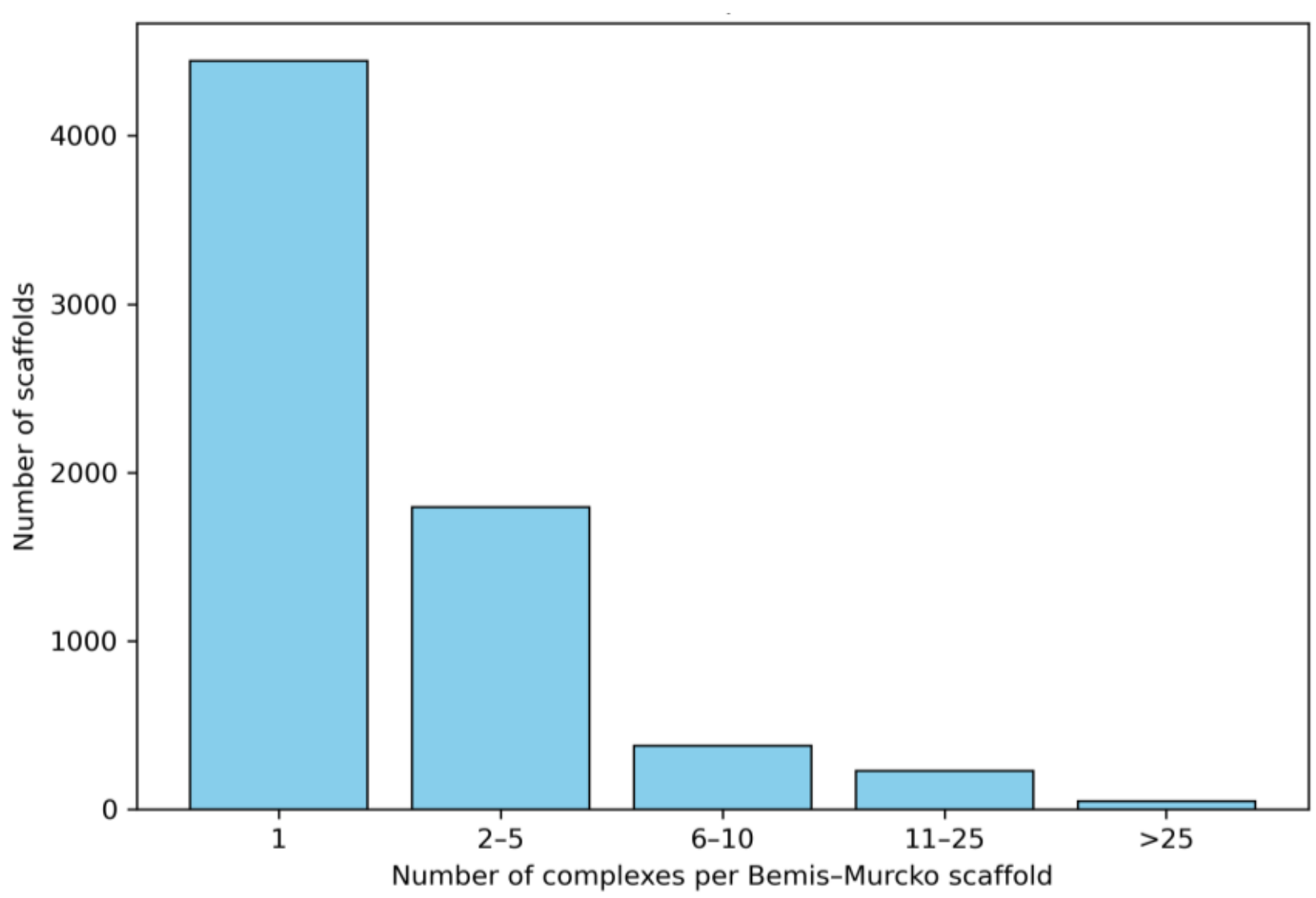

Figure S9. Chemical series and scaffold analysis of DockBind ligands. A: The distribution of number chemical series per target . Approximately 6% of the targets with at least two compounds did not contain an identified chemical series. This was generally because the compounds were small and shared only generic MCSs that occurred too frequently in ChEMBL to meet the series definition. These are listed under the category "None". In a few cases, all ligands for a target were singletons. These are also listed under the category "None". B: The distribution of representation of Bemis-Murcko scaffolds in DockBind.
